# Identification of novel rheumatoid arthritis-associated MiRNA-204-5p from plasma exosomes

**DOI:** 10.1038/s12276-022-00751-x

**Published:** 2022-03-30

**Authors:** Long-Fei Wu, Qin Zhang, Xing-Bo Mo, Jun Lin, Yang-Lin Wu, Xin Lu, Pei He, Jian Wu, Yu-Fan Guo, Ming-Jun Wang, Wen-Yan Ren, Hong-Wen Deng, Shu-Feng Lei, Fei-Yan Deng

**Affiliations:** 1 Center for Genetic Epidemiology and Genomics, School of Public Health, Medical College of Soochow University, 215123 Suzhou, Jiangsu China; 2 Jiangsu Key Laboratory of Preventive and Translational Medicine for Geriatric Diseases, Soochow University, 215123 Suzhou, Jiangsu China; 3 Department of Orthopedics, the First Affiliated Hospital of Soochow University, Suzhou, Jiangsu China; 4 Department of Rheumatology, the First Affiliated Hospital of Soochow University, Suzhou, Jiangsu China; 5 Cam-Su Genomic Resource Center, Medical College of Soochow University, 215123 Suzhou, Jiangsu China; 6 Center of Bioinformatics and Genomics, Department of Global Biostatistics and Data Science, Tulane University, New Orleans, LA USA

**Keywords:** Diagnostic markers, Non-coding RNAs

## Abstract

Rheumatoid arthritis (RA) is an autoimmune disease characterized by infiltration of immune cells in the synovium. However, the crosstalk of immune cells and synovial fibroblasts is still largely unknown. Here, global miRNA screening in plasma exosomes was carried out with a custom microarray (RA patients vs. healthy controls = 9:9). A total of 14 exosomal miRNAs were abnormally expressed in the RA patients. Then, downregulated expression of exosomal miR-204-5p was confirmed in both the replication (RA patients vs. healthy controls = 30:30) and validation groups (RA patients vs. healthy controls = 56:60). Similar to the findings obtained in humans, a decreased abundance of exosomal miR-204-5p was observed in mice with collagen-induced arthritis (CIA). Furthermore, Spearman correlation analysis indicated that plasma exosomal miR-204-5p expression was inversely correlated with disease parameters of RA patients, such as rheumatoid factor, erythrocyte sedimentation rate, and C-reactive protein. In vitro, our data showed that human T lymphocytes released exosomes containing large amounts of miR-204-5p, which can be transferred into synovial fibroblasts, inhibiting cell proliferation. Overexpression of miR-204-5p in synovial fibroblasts suppressed synovial fibroblast activation by targeting genes related to cell proliferation and invasion. In vivo assays found that administration of lentiviruses expressing miR-204-5p markedly alleviated the disease progression of the mice with CIA. Collectively, this study identified a novel RA-associated plasma exosomal miRNA-204-5p that mediates the communication between immune cells and synovial fibroblasts and can be used as a potential biomarker for RA diagnosis and treatment.

## Rheumatoid arthritis: Possible microRNA biomarker identified

A microRNA that is significantly reduced in joint tissues in rheumatoid arthritis could provide a therapeutic target and act as a biomarker for disease progression. In rheumatoid arthritis, immune cells release exosomes, tiny vesicles containing microRNA and proteins that are transferred to cells in the synovium, the connective tissue lining the inside of the joint capsule. This transfer of molecules influences synovial cell activity. Shu-Feng Lei and Fei-Yan Deng at the Medical School of Soochow University, Suzhou, China, and co-workers identifed exosomal microRNAs present in rheumatoid arthritis, and examined their effect on synovial cells. Levels of one exosomal microRNA, miR-204-5p, were significantly lower in patient samples and mice models, inversely correlating with disease severity. The team believe that chronic inflammation may suppress levels of miR-204-5p. Treatment boosting microRNA levels in mice models slowed disease progression.

## Introduction

Rheumatoid arthritis (RA) is an autoimmune disease characterized by chronic inflammation in the synovium and consequent multiple joint destruction^1^. The infiltration of immune cells has been observed in synovial membranes from individuals with RA^2^. Abnormal inflammatory activation contributes to synovial fibroblast hyperplasia, which in turn promotes cartilage degradation associated with angiogenesis^3^. Understanding the relationship between infiltrating immune cells and synovial fibroblasts would provide new diagnostic and therapeutic strategies for RA.

Extensive studies have indicated that immune cells exert effects on synovial fibroblasts mainly by secreting cytokines^4^. Inhibiting cytokines, such as tumor necrosis factor α (TNF-α), helps to improve the prognosis and prevent the progression of RA^5,6^. However, exosomes released by inflammatory cells were recently reported as another class of intercellular mediators^7,8^. Exosomes are small membrane vesicles with a diameter of 50–200 nm released from cells after fusion of multivesicular bodies with the plasma membrane^9^. These extracellular vesicles encapsulating a subset of mRNAs, noncoding RNAs, and proteins can be transferred to recipient cells and then cause local and systemic disorders. Various kinds of immune-cell-derived exosomes have been detected in body fluids, such as blood, and are being studied as ideal biomarkers in disease diagnosis or used for drug delivery vehicle development^10,11^. For example, Biro et al. observed the presence of activated complement molecules on the surface of exosomes isolated from plasma of RA patients^12^. In addition, accumulating studies have indicated that aberrant exosomal noncoding RNAs (ncRNAs) in peripheral blood are closely related to RA^13–15^. Until recently, most RA-related exosome studies have mainly focused on biomarker screening, but their origins and functions during RA pathogenesis have been less reported.

MicroRNAs (miRNAs) are a class of highly conserved short ncRNAs that regulate gene expression at the post-transcriptional level^16^. Consistent with our previous study^17^, numerous studies have found the involvement of miRNAs in immune cells in the development of RA^18,19^. In addition, miRNAs play crucial roles in the regulation of synovial fibroblast activities. For example, the inflammatory cytokine IL-17 contributes to autoimmune pathogenesis by suppressing miR-23b expression in synoviocytes^20^. Increased expression of miR-155 and miR-146 in synovial fibroblasts under inflammatory conditions was associated with the development of RA through regulation of matrix metalloproteinase and cytokine secretion^21,22^. Recent studies have also indicated that miRNAs can be packaged into exosomes and transferred to distally located cells^23,24^. Mesenchymal stem-cell-derived exosomal miR-150-5p displays therapeutic potential in RA mediated by the modulation of *MMP14* and *VEGF* expression^25^. To date, the roles of immune-cell-derived exosomal miRNAs in synovial fibroblast activation are still largely unknown.

To further explore the roles of exosomal miRNAs in RA pathogenesis, we performed comparative exosomal miRNA screening in the plasma of RA patients through a miRNA microarray. Then, downregulation of exosomal miRNA-204-5p expression was validated in two independent RA samples. In vitro, we found that T-lymphocyte-derived exosomal miRNA-204-5p can be taken up by synovial fibroblasts and then inhibits human synovial fibroblast activity. Overexpression of miRNA-204-5p suppressed synovial fibroblast activity by targeting genes related to cell proliferation and invasion. In vivo, lentivirus-mediated miR-204-5p expression attenuated disease severity in mice with collagen-induced arthritis (CIA). Our results indicated that exosomal miRNA-204-5p may be a novel molecular target for RA diagnosis and treatment.

## Materials and methods

### Study subjects

RA patients were recruited from the First Affiliated Hospital of Soochow University from December 2014 to July 2015. RA diagnosis was defined according to the 2010 American College of Rheumatology (ACR) criteria. RA patients had tender and swollen joints, and a disease activity score of 28 joints (DAS28) was recorded. Laboratory tests included C-reactive protein (CRP), rheumatoid factor (RF), cyclic citrullinated peptide antibody (CCP), and erythrocyte sedimentation rate (ESR). Peripheral blood samples were collected from each subject and stored in vacuum blood collection tubes containing sodium citrate. For healthy controls, subjects with severe cardiovascular diseases, liver and kidney dysfunction, inflammatory diseases, malignant tumors, and other immune diseases, including systemic lupus erythematosus and ankylosing spondylitis, were excluded. All the study subjects were Chinese females. The study was approved by the ethical committee of Soochow University (No: 2014-146). Written informed consent was obtained from all the subjects.

#### Discovery group

The discovery group included nine RA patients and nine age-matched healthy controls (Table 1). Plasma exosomes were isolated and mixed for each of the three samples. Finally, three pooled RA samples and three pooled control samples were generated for miRNA expression profiling through a miRNA array.

**Table 1:** Basic characteristics of the studied samples.

Variables	Discovery group		Replication group		Validation group	
	RA (*n* = 9)	Control (*n* = 9)	RA (*n* = 30)	Control (*n* = 30)	RA (*n* = 56)	Control (*n* = 60)
Age (years)	47 ± 9	48 ± 14	45 ± 10	47 ± 14	48 ± 10	70 ± 4.08^**^
Sex	Female	Female	Female	Female	Female	Female
Weight (kg)	59.2 ± 9.4	56.0 ± 5.5	54.3 ± 9.2	56.3 ± 5.8	54.6 ± 8.8	58.4 ± 6.8
Height (cm)	160 ± 5.18	159 ± 6.81	157 ± 6.10	159 ± 6.96	158 ± 6.91	156 ± 5.32
RF (IU/ml)	145.5 (3.0–494.0)	N.A.	239.9 (3.0–1110.0)	N.A.	214.7 (20.0–976.0)	N.A.
ESR (mm/h)	51.5 ± 29.3	N.A.	52.3 ± 31.2	N.A.	35.72 ± 19.7	N.A.
CCP (RU/ml)	N.A.	N.A.	N.A.	N.A.	777.98 (2.19–1608.00)	N.A.
CRP (mg/L)	22.41 (5.04–75.11)	N.A.	12.4 (1.00–75.11)	N.A.	14.76 (1.00–72.20)	N.A.
DAS28	5.237 ± 0.68	N.A.	5.47 ± 1.49	N.A.	4.92 ± 0.75	N.A.

R—association coefficient. All the data are presented as the mean ± SD or mean (range).

*RF* rheumatoid factor, *ESR* erythrocyte sedimentation rate, *CCP* cyclic citrullinated peptide antibody, *DAS28* disease activity score using 28 joint counts, *CRP* C-reactive protein. *N.A*. not applicable.

**P < 0.01.

#### Replication group

Thirty RA patients and 30 healthy controls were recruited to establish the replication group to validate potentially aberrant miRNA expression in plasma exosomes. Among them, 43 subjects—25 RA patients and 18 healthy controls—had multiple omics datasets (e.g., miRNA, DNA methylation, and mRNA) collected from peripheral blood mononuclear cells (PBMCs) we generated previously.

#### Validation group

Fifty-six RA patients and 60 healthy controls were recruited to validate the differential expression of exosomal miRNAs in plasma. The subjects enrolled were an independent population from the replication group.

### Exosomal total RNA extraction

Exosomes were isolated through a spin column-based method (Serum/Plasma Maxi Kit, Qiagen, Hilden, Germany). Briefly, plasma was initially filtered through a 0.22 μm filter to exclude cell debris before being mixed with binding buffer and added to the membrane affinity column for exosome binding. After centrifugation, the flow through was discarded, and wash buffer was added to the column to wash off nonspecific substances. Following a phenol/guanidine-based combined lysis and elution step, the sample was then applied to a silica membrane for the purification of total RNA in exosomes.

### Exosomal miRNA profiling

Based on the real-time quantitative PCR (RT-qPCR) platform, a custom microarray (CT Bioscience, Jiangsu, China) with two plates (384-well format) was designed to analyze a total of 768 miRNAs. RNA concentrations were measured with a Qubit miRNA assay, and 100 ng of RNA was reverse-transcribed to cDNA using a miRNA reverse transcription kit (CT Bioscience, Jiangsu, China) with stem-loop primers according to the manufacturer’s protocol. For normalization of possible sample-to-sample variation caused by the analysis, 1.0 pmol of synthetic *Caenorhabditis elegans* miR-39 (Qiagen, Hilden, Germany) was added to each sample as an external control. MiRNA expression was determined using a Roche LightCycler 480 II PCR system. MiRNA normalization was conducted using a robust global median normalization method or housekeeping miRNAs identified by NormFinder. Similar results were obtained with the two normalization methods. Thus, we present the data using the global median normalization method. MiRNAs with Ct values above 35 cycles were considered undetectable.

### Electron microscopy analysis

Exosome samples were absorbed onto formvar/carbon-coated nickel grids (Ted Pella, Inc., CA, USA) and incubated for 10 min before adding 2% paraformaldehyde for fixation. Later, the samples were further fixed in 2.5% glutaraldehyde and contrasted in 2% uranyl acetate. Prepared samples were observed under an electron microscope (Carl Zeiss NTS, Jena, Germany).

### NanoSight analysis

Exosome size distribution was measured by nanoparticle tracking analysis using the NanoSight system (Malvern Instruments, Malvern, UK). The device measures the Brownian motion of particles whose speed of motion, or diffusion coefficient, is related to particle size.

### Exosome uptake assay

Exosomes isolated from Jurkat cells were labeled with a PKH67 Green Fluorescent Cell Linker Kit (Sigma–Aldrich, MI, USA). Briefly, exosomes harvested from 10 cm dishes were added to 1 ml of dilute C solution before adding 4 µl of PKH67 dye to 1 ml of dilute C solution. After incubation of the exosome suspension with the dye solution, 2 ml of 1% BSA was added to stop exosome staining. The samples were then transferred to 300 kDa Vivaspin filters (GE, WI, USA) and centrifuged at 4000 × *g* for 3 min. After three washes with 5 ml of PBS, the stained sample was added to MH7A cells for 0 and 6 h. The cytoplasm and nucleus were stained with tubulin monoclonal antibody (CL594-66031, Proteintech, Hubei, China) and DAPI and then imaged with a fluorescence light microscope.

### Human T lymphocyte isolation

Peripheral blood samples were collected from eight RA patients and seven healthy controls and stored in vacuum blood collection tubes containing sodium citrate as described previously. PBMCs were isolated over a Ficoll-Hypaque gradient using Lymphoprep (Greiner Bio-one, Frickenhausen, Germany) within 4 h after phlebotomy. Then, CD3+ T cells were enriched and harvested using a human whole blood CD3-positive selection kit (StemCell Technologies, Vancouver, Canada).

### Cell culture and transfection

Human Jurkat and HEK 293 T cells were purchased from the American Type Culture Collection (ATCC). The fibroblast-like synoviocyte (FLS) cell line MH7A was prepared from intra-articular soft tissues of the knee joints of RA patients and obtained from Prof. Mei Liu (Nanjing Normal University, Jiangsu, China). Both HEK 293 T cells and MH7A cells were maintained in Dulbecco’s modified Eagle’s medium (Gibco Laboratories, NY, USA) supplemented with 10% fetal bovine serum (FBS, Biological Industries, KBH, Israel). Jurkat cells were cultured in RPMI 1640 medium (GIBCO Laboratories, NY, USA) with 10% FBS. The cell medium was supplemented with penicillin/streptomycin (TransGen, Beijing, China). All the cells were cultured at 37 °C in a humidified atmosphere of 5% CO_2_. Transfections were performed according to the manufacturer’s instructions with Lipofectamine 2000 (Invitrogen, CA, USA) or DharmaFECT Duo transfection reagent (ThermoScientific, MN, USA).

### Plasmid construction

For generation of the miR-204-5p overexpression construct, a 662-bp fragment up and downstream of human pre-miR-204 was amplified and cloned into the pCDH-GFP vector (SBI, CA, USA) using XbaI and EcoRI restriction sites. The 3′-UTR regions of *ANGPT1* and *CRKL* were amplified and then cloned into the pmirGLO vector (Promega, WI, USA), and the miR-204-5p binding site sequence was changed to GTAATCC. The primers used in this study are listed in Supplementary Table 1.

### Lentivirus package

Lentiviral vectors were based on Prof. Trono’s laboratory protocols with some modifications. The packaging plasmid pPAX2, envelope plasmid pMD2.G and pCDH plasmids (SBI, CA, USA) were transfected into HEK-293T cells with Lipofectamine 2000^26^. After 48 and 72 h of transfection, the cell culture medium containing recombinant lentiviruses was harvested and then concentrated using polyethylene glycol precipitation and centrifugation. The viral titer was determined with a qPCR Lentivirus Titration Kit (ABM, BC, Canada). Lentiviruses were added to subconfluent cultures of cells together with 8 μg/ml polybrene.

### Real-time cell proliferation assay

For MH7A cells, cell proliferation was monitored with an impedance-based instrument system (xCELLigence, CA, USA) enabling label-free real-time cell analysis. Briefly, 5 × 10^3^ cells in 100 µl of culture medium were seeded in E-Plate 96, and changes in electrical impedance are shown as a cell index that directly reflects cellular proliferation. The cell index was read automatically every 15 min, and the recorded curve is shown as the cell index ± s.e.m.

### CCK-8 assay

Jurkat cells were seeded into a 96-well plate at a density of 5 × 10^3^ cells per well. At selected time points, cell proliferation was evaluated after the addition of 10 μl of CCK-8 reagent (Dojindo, Kumamoto, Japan). Following this, the plate was incubated at 37 °C for 3 h. The absorbance at 450 nm was determined by a multimode microplate reader (BioTek, VT, USA).

### Cell viability assay

MH7A cells were seeded in 96-well plates at 5000 cells/well for 48 h. Then, a volume of CellTilter-Glo reagent (Promega, WI, USA) was added to the cell culture medium in each well. After incubation for 10 min, the luminescent signal was measured using a multimode microplate reader (BioTek, VT, USA). The cell number correlates with luminescent signal.

### EdU staining assay

EdU staining was performed with an EdU staining kit (Beyotime, Shanghai, China) according to the manufacturer’s protocol. Briefly, 2 × 10^4^ MH7A cells were seeded in 24-well plates for 48 h. After addition of EdU solution (10 µm) to the cell medium for 6 h, MH7A cells were fixed and washed three times. Then, click reaction solution including Azide 488 was added to the cultured cells for 30 min. Cell nuclei were stained with DAPI and imaged under a fluorescence microscope.

### RT-qPCR

Total RNA was extracted using TRIzol reagent (Invitrogen, CA, USA), and mRNA was transcribed into cDNA using HiScript III RT SuperMix (Novazyme, Jiangsu, China) following the manufacturer’s instructions. For miRNA, RNA was reverse-transcribed with a miRNA-specific primer by using the miScript Reverse Transcription Kit (Transgen, Beijing, China). Quantitative PCR of mRNA or miRNA was performed on an ABI Q6 Flex system (Applied Biosciences, CA, USA) with SYBR Green mix (Novazyme, Jiangsu, China). Amplification of GAPDH or RNU6 was used as a normalization control. Gene expression was calculated using the comparative Ct method.

### Flow cytometry analysis

Jurkat cells were washed three times with PBS and incubated for 30 min at 4 °C with saturating concentrations of allophycocyanin (APC)-conjugated CD69 antibody (BioLegend, CA, USA). After two washes, the cells were resuspended in PBS. Negative control cells were incubated with APC-conjugated human IgG. The immunofluorescence intensity of cells was determined by a Coulter-EPICS XL flow cytometer (Beckman, FL, USA) and analyzed using FlowJo software (Treestar, Inc., CA, USA).

### Luciferase assay

MH7A cells were cultured in six-well plates and transfected with wild-type or mutant pmirGLO luciferase reporter plasmid using DharmaFECT Duo transfection reagent. Cells were lysed 48 h post-transfection and assayed with the dual-luciferase reporter assay system (Promega, WI, USA) by a multimode microplate reader (BioTek, VT, USA). Firefly luciferase activity was normalized to Renilla luciferase activity to correct transfection efficiency variation.

### Collagen-induced arthritis (CIA) model

Animal experiments were approved by the Ethics Committee for Animals of Soochow University.

A total of 21 male DBA/1 J mice were randomly divided into four groups: CIA group (*n* = 3), CIA + PBS group (*n* = 6), CIA+lenti-NC group (*n* = 6), and CIA+lenti-204 group (*n* = 6). Eight-week-old male DBA/1 J mice were injected subcutaneously with 50 µl of bovine type II collagen (2 mg/ml, Chondrex, WA, USA) emulsified in 50 µl of complete Freund’s adjuvant containing *Mycobacterium tuberculosis* at the tail base. On Day 21 after primary immunization, mice were boosted by subcutaneous injection of 50 µl of bovine type II collagen emulsified in 50 µl of incomplete Freund’s adjuvant. On Day 30 after the onset of arthritis, mice with CIA received a total of 5 × 10^7^ transduction unit (TU) lentivirus intra-articular injections into the ankle joints. The mice in the control groups received intra-articular injections of PBS or empty lentivirus.

Mouse clinical scores were assessed every 10 days by two independent examiners, and each paw was scored for signs of arthritis on a scale of 0–4. Grade 0: no erythema and swelling; grade 1: swelling and erythema confined to the tarsals or ankle; grade 2: erythema and mild swelling extending from the ankle to the tarsals; grade 3: erythema and moderate swelling extending from the ankle to metatarsal joints; grade 4; joint rigidity. Each limb was graded, for a maximum possible score of 16 per mouse.

On Day 70 after primary immunization, the hind paw of the mice was scanned and reconstructed using a micro-CT system (SkyScan-1076, Kontich, Germany). Radiologic scores of joint destruction and bone erosion were calculated on a scale of 0–4, with higher scores indicating greater severity. Serum TNF-α was measured by ELISAs (E-EL-M0049c, Elabscience, Hubei, China) according to the manufacturer’s instructions. For histologic analysis, paws were fixed in 4% buffered formaldehyde, decalcified, embedded in paraffin, sectioned, and stained with hematoxylin and eosin, followed by CRKL (1:50, ab32018, Abcam, MA, China) and ANGPT1 (1:50, A7877, Abclonal, Hubei, China) antibodies. Histopathologic changes were scored in a blinded manner based on cell infiltration, cartilage destruction, and bone erosion parameters.

### Statistical analysis

Statistical analysis was performed using Student’s *t*-test for mean comparisons of two groups and one-way ANOVA for multiple comparisons. Spearman correlation analysis was applied to estimate the association between the levels of miR-204-5p and the common parameters for RA disease activity. *P* < 0.05 was considered statistically significant. All statistical plots were generated using GraphPad Prism 4.0 (GraphPad Software, Inc., CA, USA).

## Results

### Exosomal miR-204-5p expression is downregulated in rheumatoid arthritis (RA)

**Fig. 1:**
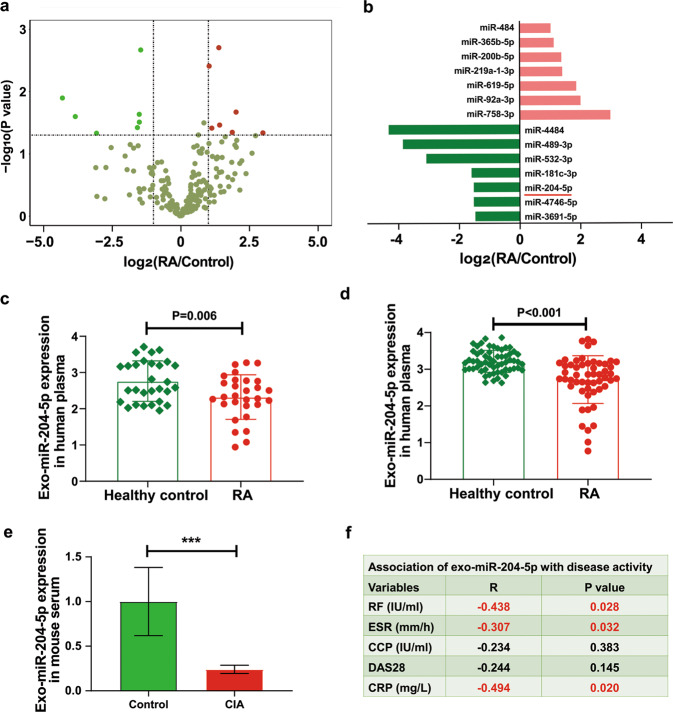
Plasma exosomal miR-204-5p expression is downregulated in rheumatoid arthritis (RA). **a** Volcano plot showing exosomal miRNAs detected by microarrays according to the fold change and *P-*value. Fold change and *P*-value were calculated by comparing the differences in exosomal miRNA between the RA patients and the healthy controls. Pink and green circles represent exosomal miRNAs with up and downregulated expression in the RA patients, respectively, (fold change > 2 and *P* < 0.05). **b** Differentially expressed exosomal miRNAs in RA displayed as a fold change versus healthy control group. **c** The expression of exosomal miR-204-5p in the replication group determined by RT-qPCR (*n* = 60, RA patients vs. healthy controls = 30:30). **d** The expression of exosomal miR-204-5p in the validation group determined by RT-qPCR (*n* = 116, RA vs. healthy controls = 56:60). **e** The expression of serum exosomal miR-204-5p in the sham group and the mice with CIA. *n* = 3 for each group. **f** Spearman linear regression analysis of exosomal miR-204-5p levels in plasma and disease activity parameters of the RA patients in the validation group (*n* = 56). Red numbers indicate statistical significance (*P* < 0.05). R represents association coefficient. Data are presented as the mean ± SD. *P*-value was calculated by Student’s *t*-test, ***P < 0.001. ESR erythrocyte sedimentation rate, CCP cyclic citrullinated peptide antibody, DAS28 disease activity score using 28 joint counts, CRP C-reactive protein.

We previously performed global miRNA expression profiling of peripheral blood mononuclear cells (PBMCs) in RA patients and identified the involvement of miRNA in peripheral immune cells during progression of RA^17^. Since immune cells release large amounts of exosomes into the blood, our further efforts contributed to identifying RA-associated miRNAs from the exosomes of immune cells. Nanosized exosomes were isolated from human plasma. Electron microscopy and nanoparticle analysis indicated that the size of the exosomes ranged from 50 to 200 nm in diameter with a rounded morphology (Supplementary Fig. 1a, b). The exosome-specific markers CD63 and CD81 were also detected by flow cytometry (Supplementary Fig. 1c, e). Next, an RT-qPCR -based microarray was applied to profile a total of 768 miRNAs in human exosomes from nine RA patients and nine sex- and age-matched controls (pooled sample for each three subjects) (Table 1). A total of 206 miRNAs (26.8%) were detected in human circulating exosomes (Fig. 1a). Among them, 7 miRNAs (miR-3691-5p, miR-4484, miR-204-5p, miR-489-3p, miR-4746-5p, miR-181c-3p, and miR-532-3p) showed significantly downregulated expression and 7 miRNAs (miR-200b-5p, miR-484, miR-92a-3p, miR-219a-1-3p, miR-365b-5p, miR-619-5p, and miR-758-3p) showed upregulated expression in RA (fold change > 2, *P* < 0.05) (Fig. 1b). Literature mining indicated that most of the identified exosomal miRNAs have been reported to participate in the pathogenesis of autoimmune diseases (Supplementary Table 2). For example, miR-92a-3p was detected in exosomes and correlated with the development of systemic lupus erythematosus (SLE)^27^.

Based on the *P*-value and exosomal expression, five miRNAs (miR-484, miR-92a-3p, miR-532-3p, miR-181c-3p, and miR-204-5p) were selected and validated in two larger independent human samples by RT-qPCR. The downregulation of exosomal miR-204-5p expression in RA was confirmed in both the replication sample (RA patients vs. healthy controls = 30:30) and validation sample (RA patients vs. healthy controls = 56:60) compared with the healthy control samples (Fig. 1c, d and Supplementary Fig. 2). Similar to the findings obtained in humans, a decreased abundance of exosomal miR-204-5p was observed in the generated mouse model of collagen-induced arthritis (CIA) (Fig. 1e). Spearman correlation analyses between exosomal miR-204-5p levels and disease activity of the RA patients in the validation group showed that exosomal miR-204-5p expression was inversely correlated with rheumatoid factor (RF) (R = −0.44, *P* = 0.028), erythrocyte sedimentation rate (ESR) (R = −0.31, *P* = 0.032), and C-reactive protein (CRP) (R = −0.49, *P* = 0.020) but not the disease activity score using 28 joint counts (DAS28) and cyclic citrullinated peptide antibody (CCP) (Fig. 1f).

### Human T lymphocytes are one source of RA-associated exosomal miR-204-5p

**Fig. 2:**
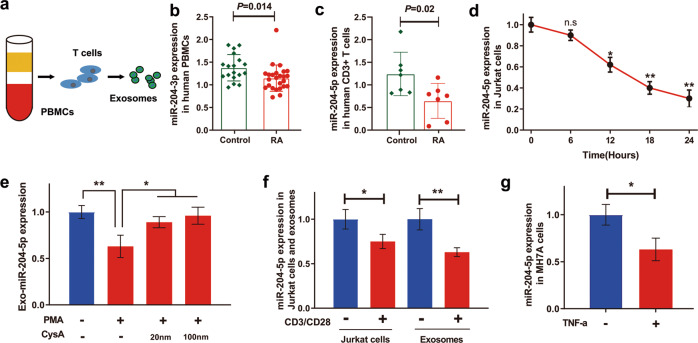
Exosomal miR-204-5p released by human T lymphocytes is decreased under inflammatory conditions. **a** Schematic representation of whole blood composition including peripheral blood mononuclear cells (PBMCs), T cells and exosomes. **b** The expression of miR-204 in PBMCs from the RA patients (*n* = 28) and the healthy controls (*n* = 15) examined by miRNA microarray. **c** The expression of miR-204-5p in CD3+ T lymphocytes in the RA patients (*n* = 7) and the healthy controls (*n* = 7) examined by RT-qPCR. **d** Human Jurkat T lymphocytes were treated with PMA (10 ng/ml), and then, the expression of miR-204-5p was determined thereafter at the indicated time points by RT-qPCR. **e** Jurkat T cells were pretreated with or without cyclosporin A (CyA) at the indicated doses (20 and 100 nM) for 30 min and then incubated with PMA (10 ng/ml) for 12 h. Exosomal miR-204-5p expression released by Jurkat T cells was determined by RT-qPCR. **f** Cells were costimulated with CD3/CD28 antibodies (2.0 µg/ml) for 24 h. MiR-204-5p expression in Jurkat T cells and exosomal miR-204-5p expression released by Jurkat T cells were then analyzed by RT-qPCR. **g** MH7A cells were treated with TNF-a (50 ng/ml) for 24 h. MiR-204-5p expression in MH7A cells was analyzed by RT-qPCR. Data are presented as the mean ± SD of three independent experiments, Student’s *t-*test in **b**,**c**, and **g**, one-way ANOVA in **e**, **f**, n.s. not significant; *P < 0.05; **P < 0.01.

PBMCs contain large amounts of circulating immune cells (e.g., T cells, B cells, dendritic cells, NK cells, and monocytes), and we hypothesized that the exosomal miR-204-5p in plasma comes from immune cells, mainly from T cells (Fig. 2a). Based on our previously generated miRNA microarray datasets of PBMCs (RA patients vs. healthy controls = 25:18)^17^, analyses of expression differences showed that only miR-204-3p expression was decreased in the RA patients (*P* = 0.014) (Fig. 2b). Given the same precursor for miR-204-5p and miR-204-3p, the instability of microarray results and heterozygosis of PBMCs, these observed results were understandable. However, these findings also provide some clues for the source of miR-204-5b. As T cells account for the largest proportion of PBMCs and aberrant T-cell activation has been recognized as the central event in chronic inflammation, we further isolated T lymphocytes from PBMCs through cell sorting in human subjects. Compared with that of the healthy controls, miR-204-5p expression was decreased in human T cells of the RA patients (RA patients vs. healthy controls = 7:7) by RT-qPCR (Fig. 2c). To test whether inflammation is responsible for the downregulated miRNA expression, we incubated human Jurkat T cells with the immune activator phorbol-12-myristate-13-acetate (PMA) to mimic inflammatory conditions in vitro. Notably, intracellular miR-204-5p expression in Jurkat T cells was reduced in a time-dependent manner (Fig. 2d). Importantly, exosomal miR-204-5p existed in the medium of Jurkat T cells cultured with exosome-depleted serum (Fig. 2e). PMA induction resulted in the downregulation of exosomal miR-204-5p expression in Jurkat T cells, while the immunosuppressive drug cyclosporin A (CysA) significantly blocked the decrease in exosomal miR-204-5p induced by PMA. This downregulation of exosomal miR-204-5p expression was also observed when we used anti-CD3/28 antibodies to induce T lymphocyte activation (Fig. 2f). Since exosomal miR-204-5p expression from T cells is downregulated under immune stimuli in vitro, human T lymphocytes may serve as one source of RA-associated exosomal miR-204-5p. In addition, miR-204-5p expression was downregulated in MH7A human synovial fibroblasts stimulated with proinflammatory TNF-α (Fig. 2g).

Next, we explored the upstream regulatory mechanisms of miR-204 through bioinformatics analysis (Supplementary Fig. 3a). Based on the previously generated methylation data^28^, the methylation status around miRNA loci was analyzed. We found that the methylation level of cg02610438 closest to the miR-204 gene was higher in the RA patients than the controls (Supplementary Fig. 3b). Moreover, miR-204 transcriptional regulatory regions were occupied by histone modification sites, such as H3K4me3, which provided favorable binding sites for transcription factors. Correlation analysis indicated that the transcription factor STAT3 level was inversely correlated with miR-204 expression (R = −0.42, *P* = 0.005) (Supplementary Fig. 3c and Supplementary Table 3). Thus, different regulatory mechanisms, such as methylation, histone modifications, and transcription factors, may jointly account for downregulated miR-204 expression in RA (Supplementary Fig. 3d).

### Exosomal miR-204-5p derived from T lymphocytes is transported into synovial fibroblasts, inhibiting cell proliferation

**Fig. 3:**
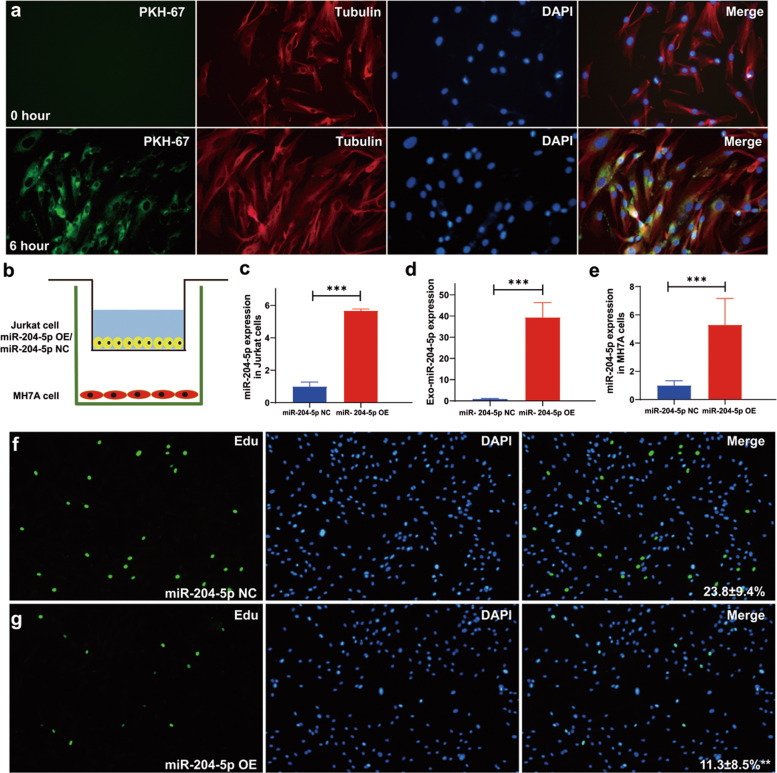
Exosomal miR-204-5p from human T lymphocytes is transferred to synovial fibroblasts. **a** Representative images of PKH67-labeled exosomes (green) from Jurkat T cells incubated with MH7A cells for 0 h (upper panel) and 6 h (lower panel) and then captured by fluorescence microscopy. Cytoplasm and nuclei were stained with tubulin (red) and DAPI (blue). **b** Schematic representation of the Transwell assay for the indicated Jurkat T cells and MH7A synovial fibroblasts. MiR-204-5p-OE, Jurkat cells transfected with miR-204-5p overexpression vector; miR-204-5p-NC, Jurkat cells transfected with miR-204-5p-negative control vector. **c**–**e** MiR-204-5p expression in Jurkat T cells, exosomes, and MH7A cells in the Transwell assay. Jurkat T cells and MH7A cells were cultured with exosome-depleted serum and examined by RT-qPCR. **f**, **g** Representative images of EdU staining in MH7A synovial fibroblasts cocultured with miR-204-5p-NC- and miR-204-5p-OE Jurkat T cells for 48 h. The relative percentage of EdU-positive MH7A cells in images of related groups is shown. Data are presented as the mean ± SD of three independent experiments. **P < 0.01.

To determine whether exosomal miR-204-5p in T lymphocytes can be taken up by synovial fibroblasts, we labeled exosomes released by Jurkat T cells with PKH67 and then added them to human MH7A synovial fibroblasts. After 6 h of incubation, MH7A cells exhibited efficient uptake of T-lymphocyte-derived exosomes, as indicated by the presence of green fluorescence staining in MH7A cells (Fig. 3a). Next, we carried out a coculture assay of MH7A and Jurkat T cells in a Transwell system with a 400 nm membrane that only allows exosomes to freely transfer (Fig. 3b). After 48 h of coculture, both the cells and supernatant exosomes were harvested, and miR-204-5p levels were examined by RT-qPCR. In the supernatant exosomes and MH7A cells, miR-204-5p was markedly elevated when MH7A cells were cocultured with the Jurkat T cells overexpressing miR-204-5p (miR-204-5p-OE) compared with the miR-204-5p-negative control cells (miR-204-5p-NC) (Fig. 3c–e). Then, we investigated the impact of exosomal miR-204-5p from T lymphocytes on synovial fibroblast proliferation through an EdU staining assay. After 48 h of coculture, the proliferation of the MH7A cells cocultured with Jurkat T cells overexpressing miR-204-5p was significantly blocked (Fig. 3f, g). When Jurkat T cells were pretreated with 10 µm GW4869, an inhibitor of exosome biogenesis and release, the suppressive effect of miR-204-5p-OE on synovial fibroblast proliferation was blocked, as indicated by EdU staining (Supplementary Fig. 4). Overall, these results demonstrated that T-lymphocyte-derived exosomal miR-204-5p can be efficiently transported into synovial fibroblasts and inhibit cell proliferation.

### Overexpression of miR-204-5p inhibits synovial fibroblast activation

**Fig. 4:**
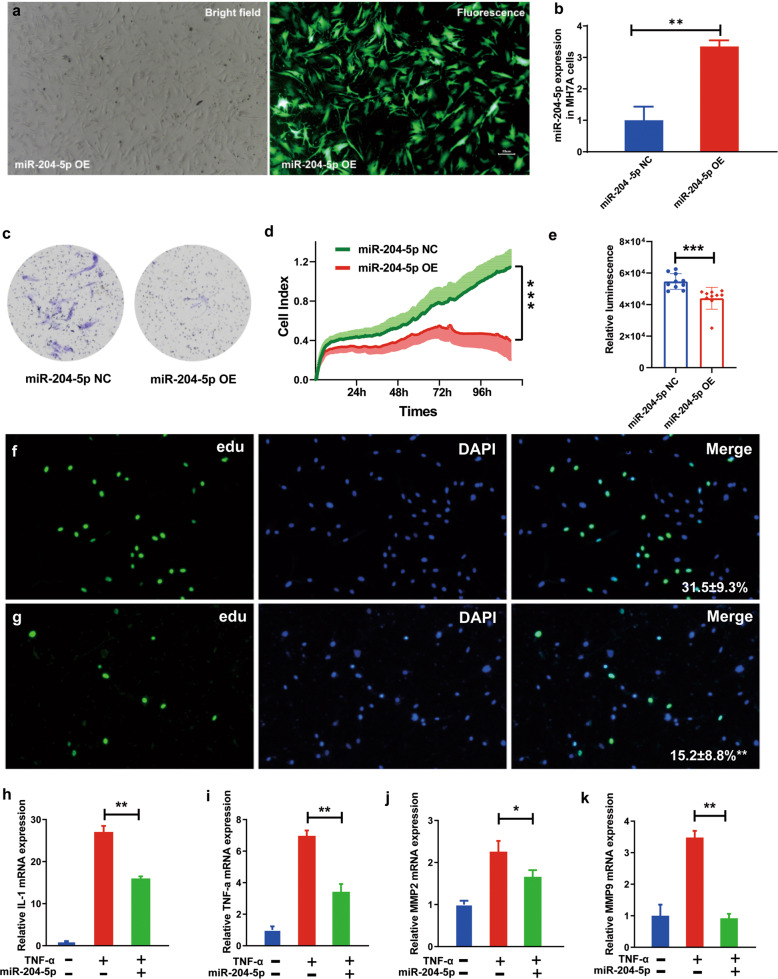
Overexpression of miR-204-5p inhibits MH7A cell invasion, proliferation, and inflammatory factor expression. **a** Representative images of miR-204-5p-OE cells in the bright field (left) and fluorescent field (right). **b** Relative expression of miR-204-5p in miR-204-5p-OE and miR-204-5p-NC cells examined by RT-qPCR. **c** MiR-204-5p-OE and miR-204-5p-NC cells from the upper well were stained with crystal violet in a cell invasion assay after culture for 48 h. **d** The cell index of miR-204-5p-OE and miR-204-5p-NC cells was recorded in real time using an xCELLigence RTCA instrument. Data are presented as the mean ± s.e.m. **e** Cell viability was examined for miR-204-5p-OE and miR-204-5p-NC cells cultured for 48 h with an ATP bioluminescence method. **f**, **g** Representative images of EdU staining for miR-204-5p-NC (**f**) and miR-204-5p-OE (**g**) cells cultured for 48 h. The relative percentage of EdU-positive MH7A cells in images of related groups is shown. **h**–**k** Inflammatory factor expression in miR-204-5p-NC and miR-204-5p-OE cells examined by RT-qPCR. Data are presented as the mean ± SD of three independent experiments, Student’s *t*-test, two-way repeated-measures ANOVA in **d**, * *P* < 0.05; **P < 0.01; ***P < 0.001.

To further determine whether the transferred exosomal miR-204-5p affects synovial fibroblasts, we constructed miR-204-5p overexpression (miR-204-5p-OE) MH7A cells through lentiviral particles (Fig. 4a, b). Synovial fibroblasts in individuals with RA exhibit cancer-cell-like characteristics; thus, a Transwell assay was applied to investigate the effect of miR-204-5p on synovial fibroblast invasion. Overexpression of miR-204-5p in MH7A cells markedly inhibited the invasive phenotype of synovial fibroblasts, as indicated by crystal violet staining, compared with that of the miR-204-5p-negative control cells (miR-204-5p-NC) (Fig. 4c). Next, we adopted impedance-based technology to monitor the effect of miR-204-5p on cell proliferation in real time. Notably, overexpression of miR-204-5p resulted in significant inhibition of synovial fibroblast proliferation (Fig. 4d). Similar results were also obtained when we carried out a luminescent cell viability assay to assess the regulatory effect of miR-204-5p on MH7A cell viability (Fig. 4e). In addition, EdU staining assays further confirmed the suppressive effect of miR-204-5p on synovial fibroblast proliferation (Fig. 4f, g). To determine whether miR-204-5p has effects on the proinflammatory reaction, we measured proinflammatory cytokine expression in the miR-204-5p-OE and miR-204-5p-NC cells through RT-qPCR. After TNF-α treatment for 48 h, the expression of proinflammatory cytokines (*IL-1* and *TNF-α*) and matrix metalloproteinases (*MMP2* and *MMP9*) was enhanced in synovial fibroblasts. However, overexpressed miR-204-5p significantly attenuated inflammatory activation, as indicated by the reduced levels of proinflammatory cytokines and matrix metalloproteinase (Fig. 4h–k). Taken together, these results suggested that miR-204-5p has suppressive effects on synovial fibroblast invasion, proliferation, and cytokine secretion.

### *CRKL* and *ANGPT1* are two target genes of miR-204-5p

**Fig. 5:**
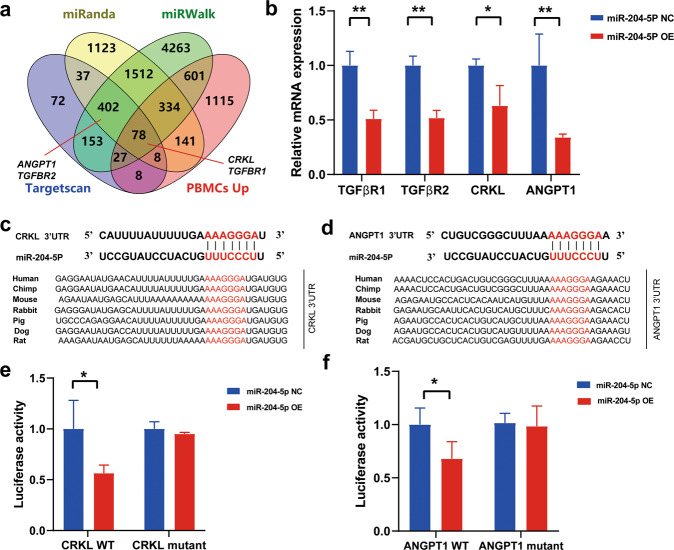
The target genes of miR-204-5p. **a** Venn diagram showing overlapping target genes for miR-204-5p predicted by the indicated software. **b** *TGFβR1, TGFβR2, CRKL*, and *ANGPT1* target gene expression in miR-204-5p-NC and miR-204-5p-OE cells using RT-qPCR. **c**, **d** Complementary sequences between miR-204-5p and the 3′-UTR of *CRKL* and *ANGPT1*. The seed sequence of miR-204-5p and the potential binding sites at the 3′-UTR of *CRKL* and *ANGPT1* are indicated in red. The binding sites of *CRKL* and *ANGPT*1 are highly conserved in mammalian species. **e**, **f** Luciferase activity determined in MH7A cells that were transfected with the indicated wild-type or point-mutated 3′ UTR reporter constructs. Data are presented as the mean ± SD of three independent experiments, Student’s *t-*test in **b** and **c**, one-way ANOVA in **e**, n.s. not significant; * *P* < 0.05; **P < 0.01.

To identify potential target genes of miR-204-5p, we identified 402 predicted targets using publicly available software, including TargetScan, miRanda and miRwalk (Supplementary Table 4). After combining 2476 genes with upregulated expression in RA based on our PBMC datasets, a total of 78 overlapping genes were obtained (Fig. 5a). Given that miR-204-5p suppressed synovial fibroblast invasion and proliferation, we selected and validated four target genes (*CRKL, ANGPT1, TGFβR1*, and *TGFβR2*), which were functionally consistent with the suppressive function of miR-204-5p in synovial fibroblasts. RT-qPCR analysis revealed that the levels of the four genes were markedly reduced in the miR-204-5p-OE cells (Fig. 5b). As *TGFβR1* and *TGFβR2* have been previously reported as targets of miR-204-5p^29^, we next mainly focused on confirming whether *CRKL* and *ANGPT1* were direct target genes of miR-204-5p. The 3′-untranslated regions (UTRs) of *CRKL* and *ANGPT1* contain putative miR-204-5p binding sites that are highly conserved across many species (Fig. 5c, d). Then, we performed luciferase activity assays with reporter cloning of the 3′-UTR sequences of *CRKL* and *ANGPT1* into the pmirGLO vector. MiR-204-5p-OE dramatically inhibited the luciferase activity of the wild-type *CRKL* and *ANGPT1* reporters, whereas the inhibitory effect of miR-204-5p was blocked in a mutation reporter that introduced several mismatch mutations into the seed sequences (Fig. 5e, f). Thus, miR-204-5p has suppressive effects on synovial fibroblast activation by targeting genes related to cell proliferation and invasion.

### Overexpression of miR-204 alleviates the severity of arthritis in mice with CIA

**Fig. 6:**
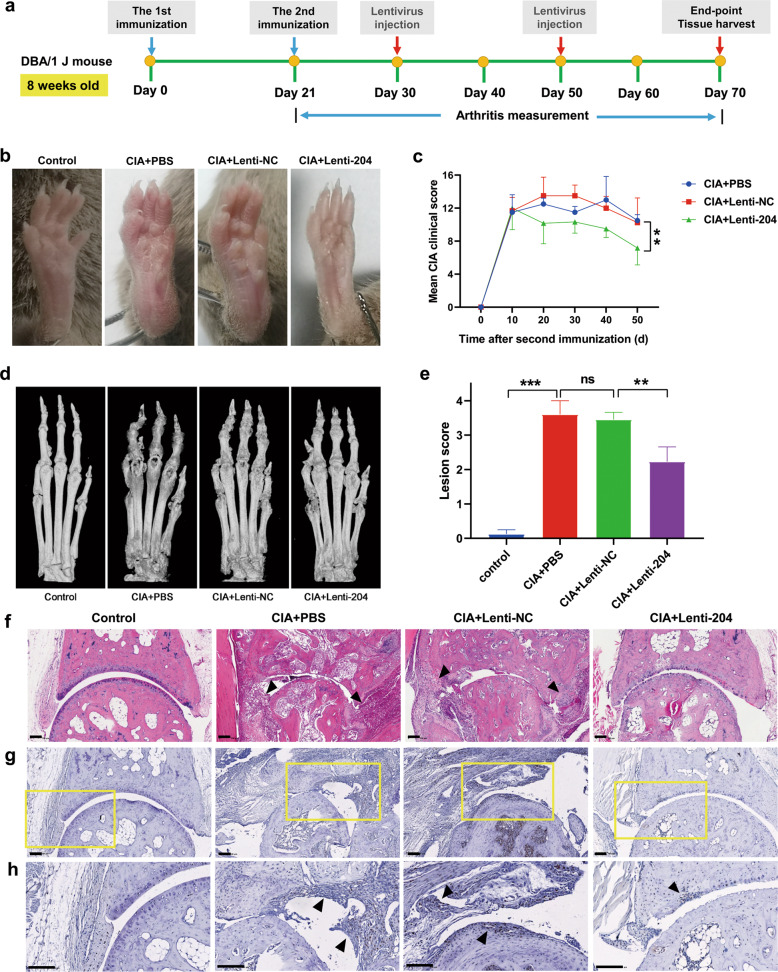
Injection of lentivirus miR-204 alleviates the disease activity of mice with collagen-induced arthritis (CIA). **a** Schematic representation of the immunization and lentiviral injection at the times indicated by the arrows with DBA/1 J mice. The mice with CIA receiving different treatments were killed on Day 70. **b** Representative images of hind paws of the mice with CIA that received the indicated PBS, lentivirus-negative control (lenti-NC) or lentivirus miR-204 overexpression (lenti-204) treatments at Day 70 after the primary immunization. *n* = 6 mice per group. **c** Mean clinical scores of the mice with CIA that received the indicated treatments were evaluated every 10 days. **d** Representative micro-CT scan photograph of the hind paws at Day 70 after the primary immunization. *n* = 6 mice per group. **e** Quantification of lesion score from **d**. **f** Hematoxylin and eosin (HE)-stained sections and evaluation of synovitis, pannus, and erosion of ankle joints in the mice with CIA 70 days after primary immunization. **g** PCNA-stained sections of ankle joints in the mice with CIA 70 days after primary immunization. **h** The enlargements of **g** indicated. Arrowheads, synovial hyperplasia. Scale bars, 100 µm. Data are presented as the mean ± SD of three independent experiments, ns not significant; **P < 0.01; ***P < 0.001 by Student’s *t*-test in **e** or two-way repeated-measures ANOVA in **c**.

In addition to the inhibitory effects on synovial fibroblasts, miR-204 overexpression resulted in the inhibition of cell proliferation (Supplementary Fig. 5a, b) and cytokine expression in T lymphocytes (Supplementary Fig. 5c, d). Therefore, we hypothesized that lentiviral-mediated miR-204 gain-of-function may be used as a novel strategy for RA treatment. To explore this hypothesis, we intra-articularly injected lentiviral vectors encoding miR-204-5p precursors into the ankle joints of the mice with CIA, and the treatment outcome was monitored by measuring the clinical, radiological, and histological changes. After arthritis was established, the mice with CIA received two lentivirus administrations (Fig. 6a). The mice treated with miR-204 overexpression lentivirus (lenti-204) exhibited less redness and swelling in the paws than the mice treated with empty lentivirus vector (lenti-NC) (Fig. 6b). Consistent with the mouse paw phenotype, arthritis scores were also reduced in the lenti-204-treated mice with CIA compared with the lenti-NC-treated mice (Fig. 6c). Image reconstructions of micro-CT demonstrated that the lenti-204-treated mice with CIA displayed less bone erosion than the lenti-NC-treated mice (Fig. 6d, e). Histopathologic examination of the ankle joints of the mice with CIA revealed the formation of synovial pannus, with synovial tissue eroding cartilage and bone. However, joint tissue displayed relatively mild changes with no erosion of cartilage in the lenti-204-treated mice (Fig. 6f). To assess synovial proliferation in the joint tissues, we stained joint tissues with proliferating cell nuclear antigen (PCNA) and found that positive staining for PCNA was significantly decreased in the miR-204 OE joint tissues (Fig. 6g, h). Moreover, we examined serum TNF-α levels through ELISAs. Compared with that of the mice treated with lenti-NC, TNF-α expression was significantly reduced in the mice injected with lenti-204 (Supplementary Fig. 6a). In addition, histological analyses of synovial tissues indicated that the expression of the miR-204 target genes *CRKL* and *ANGPT1* was reduced in the mice treated with the miR-204 overexpression lentivirus (Supplementary Fig. 6b, c). These results suggest that administration of lentiviruses expressing miR-204 markedly alleviates disease progression in mice with CIA.

## Discussion

Immune cell infiltration of the synovium is a hallmark of RA, but the roles of exosomes in cell-to-cell interactions remain poorly understood. Here, we identified a novel RA-related exosomal miR-204-5p and demonstrated the involvement of exosome-mediated transfer of miR-204-5p from T lymphocytes to synovial fibroblasts in the pathogenesis of RA. Given the easy accessibility and biological importance of this molecule, our findings provide insights into evaluating circulating exosomal miR-204-5p as a potential biomarker for RA diagnosis and treatment.

During RA progression, synovial fibroblasts are characterized by cancer-like phenotypes. Interestingly, previous evidence has shown that a lower expression level of miR-204 is correlated with various cancers, including prostate cancer^30^, gastric cancer^31^, and melanoma^32^. We found that synovial fibroblasts treated with miR-204-5p showed significant inhibition of proliferation, invasion, and inflammatory secretion. Bioinformatics analyses predicted four genes (*TGFβR1, TGFβR2, ANGPT1*, and *CRKL*) as the target genes of miR-204-5p. Among them, *TGFβR1* and *TGFβR2* are known to participate in the regulation of RA-associated pathogenic pathways^29^. In addition, RA susceptibility loci in *ANGPT1* were identified based on genome-wide association studies of RA^33^. *ANGPT1* has been reported to directly stimulate the growth and MMP-3 secretion of synovial cells by regulating the ERK/MAPK pathway^34^. Another novel target, *CRKL*, has been identified as a novel molecule for the diagnosis or treatment of RA based on computational analysis without experimental validation^35^. The present study indicated that both *ANGPT1* and *CRKL* were involved in RA, serving as direct targets of miR-204-5p in synoviocytes.

We also provided evidence for the protective effect of miR-204-5p on RA by administration of lentiviruses expressing miR-204 in the mice with CIA. Interestingly, Huang et al. recently investigated the effect of miR-204 in osteoarthritis. They also found that knockout of miR-204 in mesenchymal progenitor cells resulted in joint degeneration, while intra-articular injection of miR-204 decelerated osteoarthritic progression^36^. These data demonstrated that miR-204 functions as a suppressive factor in both mesenchymal progenitor cells and synovial fibroblasts. In contrast to their study, we provided evidence that exosomes released by immune cells, e.g., T cells, may contribute to the reduction in miR-204-5p in synovial fibroblasts. Taken together, these findings suggest that exosomal miR-204 plays essential roles in maintaining joint homeostasis to protect against RA pathogenesis. Chronic inflammation led to the reduction of exosomal miR-204 released by immune cells. The suppressive effect of miR-204 was diminished due to decreased exosomal miR-204 received in synovial fibroblasts, which in turn accelerated cell proliferation, invasion, and cytokine release.

To our knowledge, both systemic and local transfer with viral vectors have been successfully used in RA treatment. Herein, we adopted an approach through direct intra-articular injection of ankle joints using lentiviral vectors. Direct intra-articular injection could reduce exposure of extraarticular tissues, thereby avoiding deleterious side effects associated with systemic therapy to the greatest extent. In addition, systemic administration often results in short-term serum levels of expressed molecules^37,38^. Thus, a relatively high dosage or repeated injections are required to achieve constant therapeutic concentrations in the inflamed joints. For chronic and relapsing diseases such as RA, local administration provides a more efficient, tissue-specific transduction strategy. However, our results suggested that the antiarthritic effects of local intra-articular injection with lentivirus vector probably have potential systemic anti-inflammatory effects, which was evidenced by the significantly reduced TNF-α expression in the mice injected with lenti-miR-204 (Supplementary Fig. 6a). This phenomenon has also been observed in several studies across different animal models of RA^39–45^. Bakker et al. first described that transplantation of cells producing an interleukin-1 receptor antagonist to the knee cavity prevented the onset of CIA not only in the knee but also in the ipsilateral paw^46^. Similarly, in vivo periarticular gene therapy with IL-10 to the selected joints can prevent peripheral nontreated joints from developing CIA^38^. To date, the exact mechanism of this effect remains unclear. Cell trafficking studies indicated that leucocytes or fibroblasts may account for this effect. Experiments with intra-articular injection of adenoviral vector containing luciferase as a marker indicated that leukocytes in injected joints could migrate to the adjacent lymph node and opposing inflamed joints^38^. Another possible mechanism may involve exosomes derived from dendritic cells. Kim et al. found that periarticular administration of exosomes derived from IL-10-treated dendritic cells could suppress inflammation within injected and untreated contralateral joints^45^. In addition, neurogenic factors, such as substance P or corticotropin-releasing hormone, may be responsible for this systemic effect^47^. Thus, the antiarthritic protective effects of local intra-articular therapy may not be limited to the target joint but can affect distal joints.

However, some potential limitations still exist in this study. First, we adopted direct injection of overexpression miR-204 vector for RA treatment instead of injecting exosomes with miR-204. Second, this study investigated the communication from immune cells to synovial fibroblasts mediated by exosomes. In contrast, a previous study also reported communication from synovial fibroblasts to immune cells; synovial fibroblast-derived exosomes carrying TNF-α were shown to be effectively transferred to activated T cells and render activated T cells resistant to apoptosis^10^. Thus, the exosome-mediated dialog between immune cells and synovial fibroblasts in both directions requires further attention. Third, this study mainly focused on miR-204-5p transfer from T cells to synoviocytes. However, other immune cells from peripheral blood probably release exosomes to control synovial fibroblast activation. To our knowledge, miR-204-5p is present in exosomes derived from multiple cells, such as mesenchymal stem cells and vascular smooth muscle cells^48,49^. In addition, miR-204-5p is expressed in joint chondrocytes and immune cells, such as B cells, dendritic cells, and macrophages^36,50–54^. For example, miR-204-5p was aberrantly expressed in activated dendritic cells and contributed to immune infiltration^50^. Increased expression of miR-204-5p in B cells was also related to the clearance of bacterial infection^51^. Therefore, these cells provide potential origins of exosomal miR-204-5p. To date, it has been challenging to precisely measure the amount and composition of exosomes, which exhibit dynamic changes as disease progresses. With the rapid development of cutting-edge technological tools, such as single-cell sequencing, we believe that it would help to elucidate the mechanisms governing the origin of exosomes in the niches of local inflammatory lesions in RA.

In conclusion, this study enhanced our understanding of exosome-mediated immune cell and synovial fibroblast crosstalk and highlighted the essential roles of miR-204-5p in maintaining joint homeostasis and protecting against RA progression. The main strength of this study was to elucidate the roles of exosomal miR-204-5p in RA by taking advantage of a human cohort, animal model, and human cell lines. Such an interdisciplinary research strategy not only enables us to identify novel RA-related exosomal miR-204-5p but also helps us to systematically investigate its biological functions in RA pathogenesis.

## Supplementary information

Supplemental information

## Data Availability

Data are available on reasonable request.
