# Supplementary material for: Identification of novel rheumatoid arthritis-associated MiRNA-204-5p from plasma exosomes
Source: Exp Mol Med. 2022 Mar 30;54(3):334–45. doi: 10.1038/s12276-022-00751-x (PMC8980013; doi:10.1038/s12276-022-00751-x)
Supplement: Supplementary file 1 — Supplemental information [file 12276_2022_751_MOESM1_ESM.pdf]

Supplementary Figures

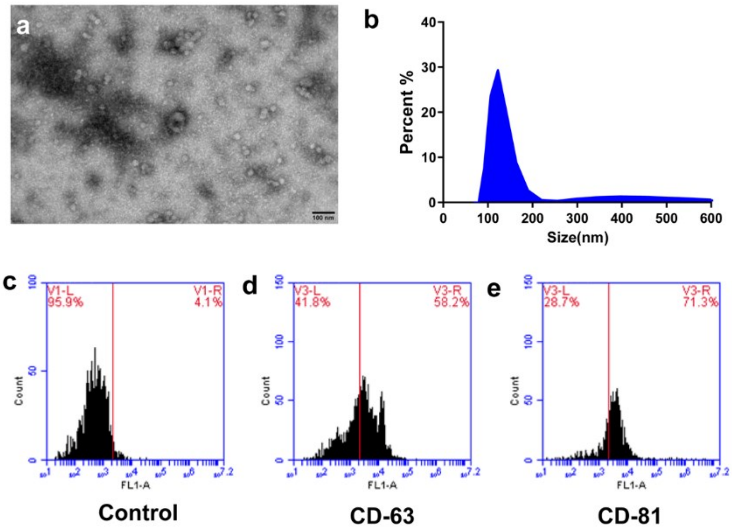

Supplementary Fig. 1. Characterization of the exosomes isolated from plasma. (a) Representative images of exosomes size and shape visualized by transmission electron microscopy (TEM). **(b)** Analysis of the size distribution of the exosomes isolated from plasma using the NanoSight technology. **(c-e)** Representative images of flow cytometry analyses the percentage of CD63+ and CD81+ exosomes harvested from healthy controls. Control, isotype-matched control antibody. Scale bar, 100 um. Data are presented as images of at least three independent experiments

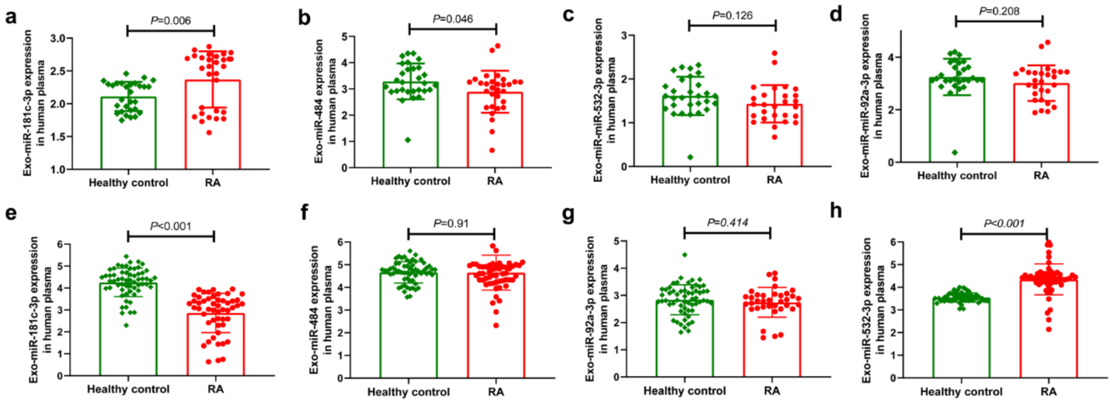

Supplementary Fig. 2. The expression of identified exosomal miRNAs in validation and replication group. (a-d) The expression of exosomal miR-484, miR-92a-3p, miR-532-3p, and miR-181c-3p in replication group measured by RT-qPCR (n=60, RAs vs. healthy controls=30:30). **(e-h)** The expression of exosomal miR-484, miR-92a-3p, miR-532-3p, and miR-181c-3p in validation group measured by RT-qPCR (n=116, RAs vs. healthy controls=56:60). Data are presented as Mean ± SD, Student's *t* test with *P* value indicated.

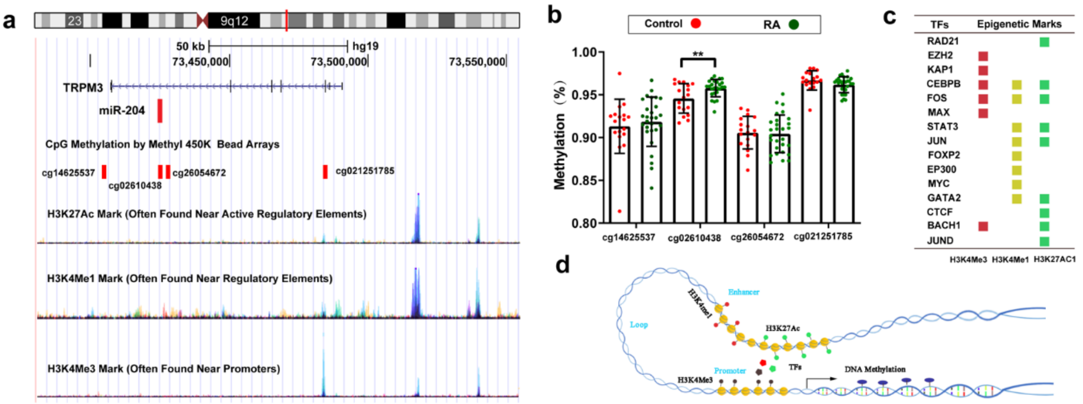

Supplementary Fig. 3. Upstream regulatory mechanisms jointly account for miR-204 downregulation in RA. (a) Structure of miR-204-5p gene, DNA methylation sites derived from Methyl 450K Bead Array, histone modification patterns (H3K4me3, H3K4me1 and H3K27ac) from ENCODE project are viewed in the UCSC genome browser. The histone modification track data from ChIP-seq assay uses a transparent overlay method to display results from a number of cell lines with a particular color. The image was generated on a customized installation of the UCSC Genome Browser. Red line represents the location of miR-204-5p in chromosome. **(b)** The methylation signals differences for 4 CpG sites around miR-204 gene locus examined by 450K Bead Array between RA (n=25) and healthy controls (n=18) in methylation datasets we generated previously[1]. **(c)** Histone modifications locus occupied by specific Transcription factors predicted by ENCODE project. **(d)** Schematic overview of miR-204 regulation elements including histone modifications (H3K4me3, H3K4me1 and H3K27ac), DNA methylation and specific transcription factors. Data are presented as Mean ± SD, Student's *t* test, **, P<0.01.

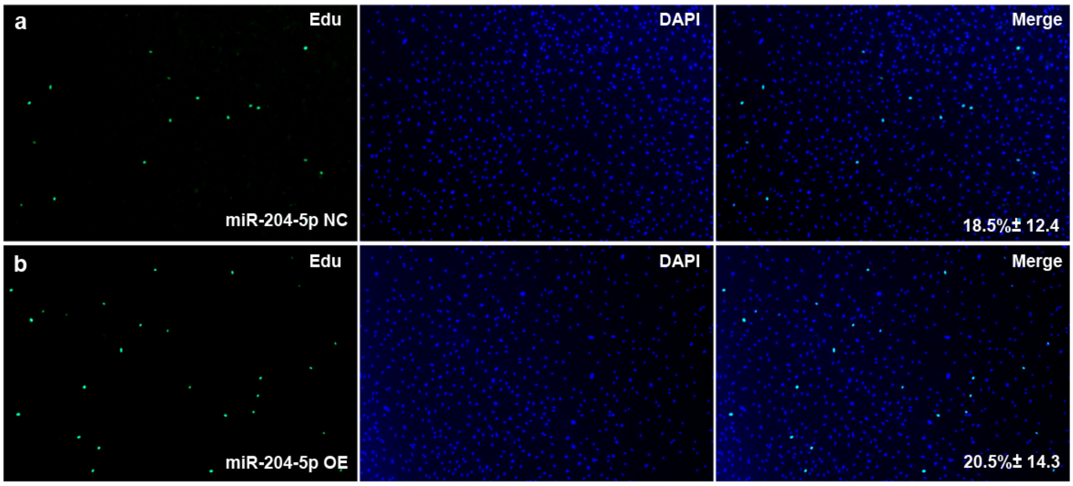

Supplementary Fig. 4. Inhibition of exosomes release block the suppressive effect of miR-204-5p OE on synovial fibroblasts proliferation. Jurkat T cells overexpression of miR-204-5p (a) or negative control vector (b) were treated with GW4869 (10 um). After 24hr, exosomes of the Jurkat T cells were collected before incubation with human synovial fibroblasts MH7A cells. After 48 hours incubation, MH7A cell proliferation was measured through Edu assay.

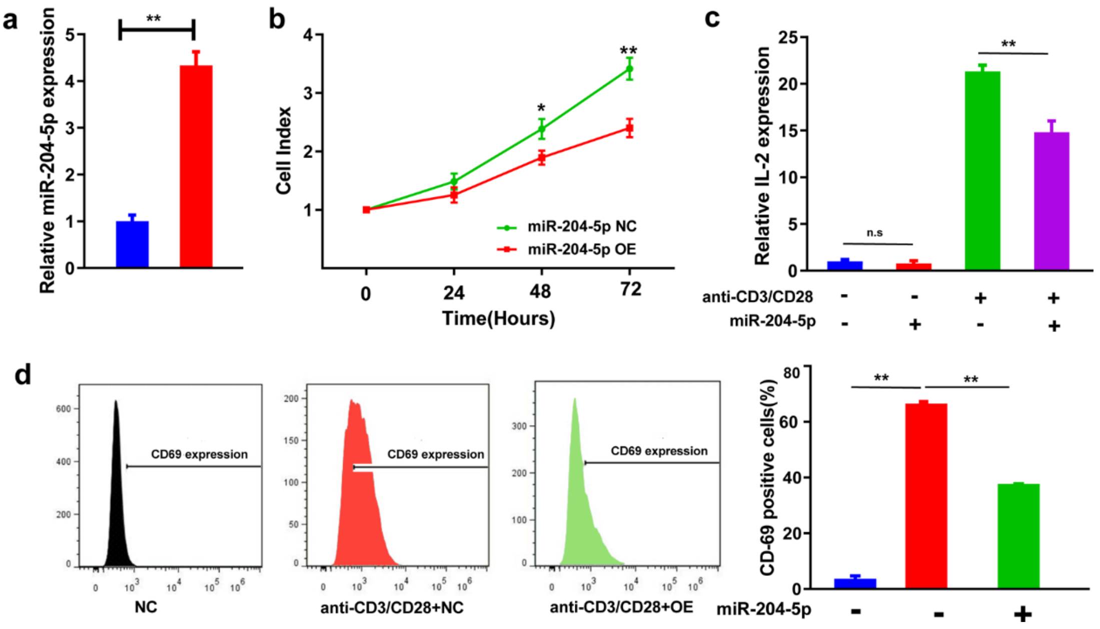

Supplementary Fig. 5. miR-204-5p negatively regulates human T-cell activation. (a) miR-204-5p expression in the miR-204-5p OE and NC cell lines examined by RT-qPCR. Jurkat cells stably transfected with miR-204-5p (OE) or negative control (NC). **(b)** Cell numbers in miR-204-5p OE and NC cell lines determined by CCK-8 assay. **(c)** IL-2 expression was examined by RT-qPCR in miR-204-5p OE and NC cells treated with CD3/CD28 antibodies (2.0 ug/ml) for 24 hours. **(d)** Surface CD69 expression was determined by flow cytometry in miR-204-5p OE and NC cells treated with CD3/CD28 antibodies (2.0 ug/ml) for 24 hours. Right panel: qualitative analysis of CD-69 positive cells. Data are presented as Mean ± SD of three independent experiments, Student's *t*-test in a, One-way ANOVA in b-d, ns, not significant; * $P < 0.05$; ** $P < 0.01$.

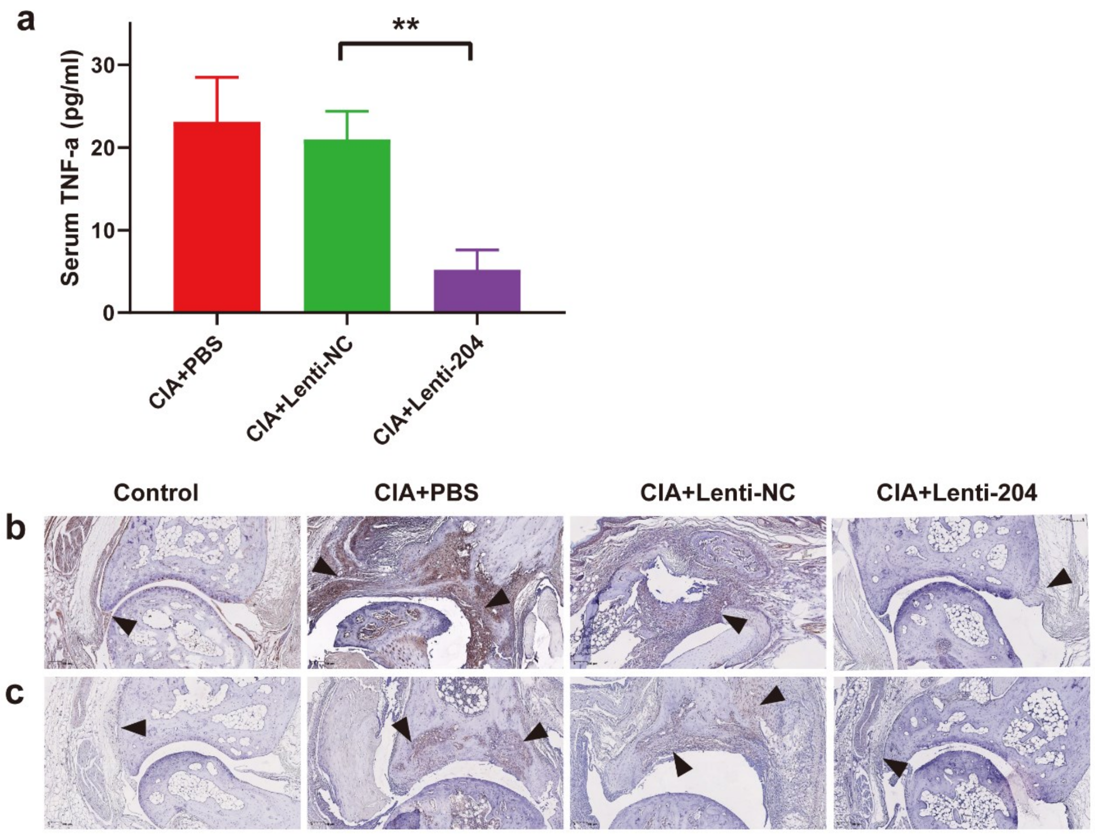

Supplementary Fig. 6. Injection of lentivirus miR-204 alleviates disease activity of collagen-induced arthritis (CIA) mice. (a) CIA mice receiving different treatments were killed on day 70 and serum TNF-α level was examined by ELISA assay. n=6 mice per group. **(b-c)** Representative ANGPT1 (b) and CRKL(c)-stained sections of ankle joints in CIA mice 70 days after primary immunization. Arrowheads, synovial tissue. Data are presented as Mean ± SD. One-way ANOVA in a; **$P < 0.01$.

Supplementary Tables

Supplementary Table 1. The primers used in this study

Primer	Sequence (5'to3')
**RT-qPCR primers**	
IL-2-forward	CATTGCACTAAGTCTTGCACTTGTCA
IL-2-reverse	CGTTGATATTGCTGATTAAGTCCCTG
CRKL-forward	TCTTTGCGAAAGCAATCCAG
CRKL-reverse	TCACGATGTCACCAACCTC
ANGPT1-forward	CTAGATTTCCAAAGAGGCTGG
ANGPT1-reverse	ATTCACCGGAGGGATTTCC
TGFBR1-forward	AGCTGTGAAGCCTTGAGAG
TGFBR1-reverse	CAATGCTGTAAGCCTAGCTG
TGFBR2-forward	AATCCTGCATGAGCAACTG
TGFBR2-reverse	CATTCTTTCTCCATACAGCCA
TNF-α-forward	GGCTCCAGGCGGTGCTTGTTC
TNF-α-reverse	AGACGGCGATGCGGCTGATG
IL-1β-forward	ATGGCAGAAGTACCTAAGCTCGC
IL-1β-reverse	ACACAAATTGCATGGTGAAGTCAGTT
GAPDH-forward	GAAGGTGAAGGTCGGAGT
GAPDH-reverse	CTTCTACCACTACCCTAAAG
**Luciferase Reporter primers:**	
ANGPT1 UTR-forward：	GG GTTTAAAC GAAAGCAACAAAGAAATCCG
ANGPT1 UTR-reverse：	GCTCTAGA ACCACATACATCCTTACTTG
CRKL UTR-forward：	GG GTTTAAAC AGGGATACTCTGTTTTTCAC
CRKL UTR-reverse：	GCTCTAGA TTAGAAAACAGAACCAGCAC
ANGPT1 UTR mutation Left-forward：	GGGTTTAAAC GAAAGCAACAAAGAAATCCG
ANGPT1 UTR mutation Left-reverse：	GCAGTTTCTTCCCTTTTTAAAGCCCGACAGTCAGTGGAGTTTTCTA
ANGPT1 UTR mutation Right-forward：	GGCTTTAAAAAGGGAA GAAACTGCTGAGCTTGCTGTGCTTCAAAC
ANGPT1 UTR mutation Right-reverse：	GCTCTAGAACCACATACATCCTTACTTG
CRKL UTR mutation Left-forward：	GG GTTTAAAC AGGGATACTCTGTTTTTCAC
CRKL UTR mutation Left-reverse：	AAACCACATCAGGATTACTCAAAAATAAAATGTTCATATTCCTC
CRKL UTR mutation Right-forward：	ATTTTTGAGTAATCCTGATGTGGTTTTTTGCCAGGTGTTTATAATTA
CRKL UTR mutation Right-reverse：	GCTCTAGA TTAGAAAACAGAACCAGCAC
**miR-204 overexpression vector primers:**	
miR-204 -forward：	GC TCTAGA GAAGAAGATGGTGGTTAGTT
miR-204-reverse：	G GAATTC ATCTCTCTTATGGGACAGTT

Supplementary Table 2. Distributions and known functions of the exosomal miRNAs in the RA pathogenesis

miRNAs	FC	*P*	Accession Number	Known Distributions	Disease
**Increased miRNAs in RA**					
hsa-miR-200b-5p	2.61	0.002	MIMAT0004571	Tumor tissues, Plasma, Exosome	Cancer
hsa-miR-484	2.04	0.004	MIMAT0002174	Tumor tissues, Plasma, Urinary	Cancer
hsa-miR-92a-3p	4.03	0.021	MIMAT0000092	T cells, Plasma, Exosome	Immune disease
hsa-miR-219a-1-3p	2.66	0.034	MIMAT0004567	Unknown	Unknown
hsa-miR-365b-5p	2.18	0.039	MIMAT0022833	Plasma	Obese, cancer
hsa-miR-619-5p	3.66	0.045	MIMAT0026622	Plasma	Cancer
hsa-miR-758-3p	7.93	0.046	MIMAT0003879	Spleen, Plasma	SLE
**Decreased miRNAs in RA**					
hsa-miR-3691-5p	-2.76	0.002	MIMAT0018120	Unknown	Unknown
hsa-miR-4484	-19.94	0.013	MIMAT0019018	Tumor tissues, salivary, exosome	Cancer
hsa-miR-204-5p	-2.86	0.023	MIMAT0000265	T cells, Tumor cells, Bone	T-cell activation, Cancer
hsa-miR-489-3p	-14.40	0.025	MIMAT0002805	Bone, Plasma, Tumor cells	Bone metabolism, Cancer
hsa-miR-4746-5p	-2.86	0.031	MIMAT0019880	Unknown	Unknown
hsa-miR-181c-3p	-3.00	0.038	MIMAT0004559	Serum, T cells, Exosome	T cell activation, Cancer
hsa-miR-532-3p	-8.43	0.047	MIMAT0004780	Tumor tissues, Macrophage cells	Cancer, Inflammatory

Supplementary Table 3. Association of transcription factor and miR-204 expression in PBMCs

TF	H3K4Me3	H3K4Me1	H3K27Ac	R	*P*
**CTCF**			○	-0.214	0.168
**STAT3**		○	○	**-0.420**	**0.005**
**JUN**		○	○	0.146	0.350
**FOS**	○	○	○	-0.068	0.666
**EP300**			○	-0.300	0.051
**MYC**		○		-0.249	0.108
**BACH1**	○		○	-0.281	0.068
**MAX**	○			-0.034	0.829
**RAD21**			○	0.038	0.808
**JUND**			○	-0.175	0.263
**EZH2**	○			0.157	0.315
**GATA2**		○	○	-0.082	0.600
**CEBPB**	○	○	○	-0.167	0.285
**MYC**		○		-0.004	0.979

Notes: Hollow circle represents presence of TFs binding sites at the corresponding histone modifications marks. Significant correlation between TFs and miR-204-5p expression in PBMCs was highlighted in bold. R, association coefficient. TF, transcription factor. The correlation analysis was performed in 43 subjects. Each subject has both transcriptome-wide mRNA and miRNA expression data from PBMCs.

Supplementary Table 4a: 402 target genes overlapped in "Targetscan", "miRanda" and "miRWalk"

Target gene	Representative transcript	Gene name
RAB22A	ENST00000244040.3	RAB22A, member RAS oncogene family
SAMD5	ENST00000367474.1	sterile alpha motif domain containing 5
PHOX2B	ENST00000226382.2	paired-like homeobox 2b
DRAP1	ENST00000312515.2	DR1-associated protein 1 (negative cofactor 2 alpha)
MRPS17	ENST00000426595.1	28S ribosomal protein S17, mitochondrial; HCG1984214, isoform CRA_a
TPT1	ENST00000379056.1	tumor protein, translationally-controlled 1
MAPRE2	ENST00000285298.4	mitochondrial ribosomal protein S17
FAM160A2	ENST00000436190.2	microtubule-associated protein, RP/EB family, member 2
C10orf11	ENST00000449352.2	family with sequence similarity 160, member A2
EPHB6	ENST00000496424.2	chromosome 10 open reading frame 11
ELOVL6	ENST00000392957.2	EPH receptor B6
CAMK1	ENST00000394607.3	ELOVL fatty acid elongase 6
XKR9	ENST00000256460.3	calcium/calmodulin-dependent protein kinase I
UFD1L	ENST00000408926.3	XK, Kell blood group complex subunit-related family, member 9
EPHA7	ENST00000399523.1	ubiquitin fusion degradation 1 like (yeast)
SRP19	ENST00000369303.4	EPH receptor A7
HSD17B2	ENST00000282999.3	signal recognition particle 19kDa
SSRP1	ENST00000199936.4	hydroxysteroid (17-beta) dehydrogenase 2
NXPH4	ENST00000278412.2	structure specific recognition protein 1
B3GNT5	ENST00000349394.5	neurexophilin 4
NDRG3	ENST00000326505.3	UDP-GlcNAc:betaGal beta-1,3-N-acetylglucosaminyltransferase 5
NRBF2	ENST00000373803.2	NDRG family member 3
EPHA5	ENST00000277746.6	nuclear receptor binding factor 2
RIMS2	ENST00000273854.3	EPH receptor A5

BIN1	ENST00000507740.1	regulating synaptic membrane exocytosis 2
SFXN2	ENST00000376113.2	bridging integrator 1
SDHAF1	ENST00000369893.5	sideroflexin 2
DHH	ENST00000378887.2	succinate dehydrogenase complex assembly factor 1
SOX4	ENST00000266991.2	desert hedgehog
DVL3	ENST00000244745.1	SRY (sex determining region Y)-box 4
AP2A2	ENST00000313143.3	dishevelled segment polarity protein 3
JRKL	ENST00000448903.2	adaptor-related protein complex 2, alpha 2 subunit
AP1S1	ENST00000458427.1	jerky homolog-like (mouse)
ZNF521	ENST00000337619.5	adaptor-related protein complex 1, sigma 1 subunit
RSPO4	ENST00000361524.3	zinc finger protein 521
ALPL	ENST00000217260.4	R-spondin 4
SF3B1	ENST00000374840.3	alkaline phosphatase, liver/bone/kidney
HMGA2	ENST00000335508.6	splicing factor 3b, subunit 1, 155kDa
ANKRD13C	ENST00000403681.2	high mobility group AT-hook 2
PDE3A	ENST00000370944.4	ankyrin repeat domain 13C
HELLS	ENST00000359062.3	phosphodiesterase 3A, cGMP-inhibited
ST7	ENST00000394036.1	helicase, lymphoid-specific
SMOC1	ENST00000393451.3	suppression of tumorigenicity 7
C21orf33	ENST00000381280.4	SPARC related modular calcium binding 1
CCNY	ENST00000291577.6	chromosome 21 open reading frame 33
FOXC1	ENST00000374706.1	cyclin Y
TTYH1	ENST00000380874.2	forkhead box C1
DNM2	ENST00000301194.4	tweety family member 1
SEC61A2	ENST00000314646.5	dynamin 2
SLC43A1	ENST00000379020.4	Sec61 alpha 2 subunit (S. cerevisiae)

CAMK2D	ENST00000278426.3	solute carrier family 43 (amino acid system L transporter), member 1
EFNB3	ENST00000296402.5	calcium/calmodulin-dependent protein kinase II delta
MMGT1	ENST00000226091.2	ephrin-B3
SLC35B3	ENST00000305963.2	membrane magnesium transporter 1
TMOD3	ENST00000379660.4	solute carrier family 35 (adenosine 3'-phospho 5'-phosphosulfate transporter), member B3
NOVA1	ENST00000308580.7	tropomodulin 3 (ubiquitous)
TOX3	ENST00000465357.2	neuro-oncological ventral antigen 1
ACADL	ENST00000407228.3	TOX high mobility group box family member 3
SASS6	ENST00000233710.3	acyl-CoA dehydrogenase, long chain
TMBIM1	ENST00000287482.5	spindle assembly 6 homolog (C. elegans)
DPF1	ENST00000258412.3	transmembrane BAX inhibitor motif containing 1
VHL	ENST00000420980.2	D4, zinc and double PHD fingers family 1
ANGPT1	ENST00000256474.2	von Hippel-Lindau tumor suppressor, E3 ubiquitin protein ligase
RHOBTB1	ENST00000520734.1	angiopoietin 1
CTNNBIP1	ENST00000337910.5	Rho-related BTB domain containing 1
EZR	ENST00000377263.1	catenin, beta interacting protein 1
SSR3	ENST00000337147.7	ezrin
HSPH1	ENST00000476217.1	signal sequence receptor, gamma (translocon-associated protein gamma)
NCOA7	ENST00000320027.5	heat shock 105kDa/110kDa protein 1
KHDRBS3	ENST00000392477.2	nuclear receptor coactivator 7
ZCCHC24	ENST00000355849.5	KH domain containing, RNA binding, signal transduction associated 3
EPHB2	ENST00000372336.3	zinc finger, CCHC domain containing 24
PRRX1	ENST00000374632.3	EPH receptor B2
MTMR7	ENST00000367760.3	paired related homeobox 1
CA11	ENST00000180173.5	myotubularin related protein 7
RAP2C	ENST00000084798.4	carbonic anhydrase XI

C16orf72	ENST00000342983.2	RAP2C, member of RAS oncogene family
SGIP1	ENST00000327827.7	chromosome 16 open reading frame 72
PPP3R1	ENST00000371036.3	SH3-domain GRB2-like (endophilin) interacting protein 1
AP3M1	ENST00000234310.3	protein phosphatase 3, regulatory subunit B, alpha
SLC25A35	ENST00000355264.4	adaptor-related protein complex 3, mu 1 subunit
NEUROG2	ENST00000577745.1	solute carrier family 25, member 35
ZNF629	ENST00000313341.3	[illegible]eurogenin 2
CCND2	ENST00000262525.4	zinc finger protein 629
EFEMP2	ENST00000261254.3	cyclin D2
DUSP19	ENST00000307998.6	EGF containing fibulin-like extracellular matrix protein 2
SDHD	ENST00000354221.4	dual specificity phosphatase 19
COL5A3	ENST00000526592.1	succinate dehydrogenase complex, subunit D, integral membrane protein
SOX11	ENST00000264828.3	collagen, type V, alpha 3
RNF170	ENST00000322002.3	SRY (sex determining region Y)-box 11
ISM1	ENST00000534961.1	ring finger protein 170
NR4A2	ENST00000262487.4	isthmin 1, angiogenesis inhibitor
ATP13A4	ENST00000339562.4	nuclear receptor subfamily 4, group A, member 2
HAPLN1	ENST00000400270.2	ATPase type 13A4
CBFA2T3	ENST00000274341.4	hyaluronan and proteoglycan link protein 1
ARX	ENST00000327483.5	core-binding factor, runt domain, alpha subunit 2; translocated to, 3
TGFBR2	ENST00000379044.4	aristaless related homeobox
HGSNAT	ENST00000359013.4	transforming growth factor, beta receptor II (70/80kDa)
DNAJB4	ENST00000379644.4	heparan-alpha-glucosaminide N-acetyltransferase
ELAVL3	ENST00000370763.5	DnaJ (Hsp40) homolog, subfamily B, member 4
PALM2-AKAP2	ENST00000359227.3	ELAV like neuron-specific RNA binding protein 3
BCL2L2	ENST00000374530.3	PALM2-AKAP2 readthrough

JPH3	ENST00000250405.5	BCL2-like 2
NBR1	ENST00000284262.2	junctophilin 3
CSRNP2	ENST00000542611.1	neighbor of BRCA1 gene 1
HOXC8	ENST00000228515.1	cysteine-serine-rich nuclear protein 2
GCNT2	ENST00000040584.4	homeobox C8
EDEM1	ENST00000316170.3	glucosaminyl (N-acetyl) transferase 2, I-branching enzyme (I blood group)
RUNX2	ENST00000256497.4	ER degradation enhancer, mannosidase alpha-like 1
POU3F2	ENST00000371432.3	runt-related transcription factor 2
RNF217	ENST00000328345.5	POU class 3 homeobox 2
RHOBTB3	ENST00000521654.2	ring finger protein 217
FBN2	ENST00000379982.3	Rho-related BTB domain containing 3
PLAG1	ENST00000262464.4	fibrillin 2
AFAP1	ENST00000316981.3	pleiomorphic adenoma gene 1
MANEAL	ENST00000360265.4	actin filament associated protein 1
MCCD1	ENST00000397631.3	mannosidase, endo-alpha-like
FGF18	ENST00000376191.2	mitochondrial coiled-coil domain 1
TMEM87B	ENST00000274625.5	fibroblast growth factor 18
ATF2	ENST00000283206.4	transmembrane protein 87B
FJX1	ENST00000487334.2	activating transcription factor 2
ARHGAP30	ENST00000317811.4	four jointed box 1 (Drosophila)
LRRC8D	ENST00000368016.3	Rho GTPase activating protein 30
SERINC3	ENST00000337338.5	leucine rich repeat containing 8 family, member D
HAS2	ENST00000342374.4	serine incorporator 3
TPRG1L	ENST00000303924.4	hyaluronan synthase 2
ARHGAP10	ENST00000378344.2	tumor protein p63 regulated 1-like
TCF4	ENST00000336498.3	Rho GTPase activating protein 10

REEP1	ENST00000354452.3	transcription factor 4
AGBL4	ENST00000165698.5	receptor accessory protein 1
SLA2	ENST00000371839.1	ATP/GTP binding protein-like 4
PDE4A	ENST00000262866.4	Src-like-adaptor 2
RTKN2	ENST00000380702.2	phosphodiesterase 4A, cAMP-specific
MTMR6	ENST00000373789.3	rhotekin 2
CCDC40	ENST00000381801.5	myotubularin related protein 6
CDH4	ENST00000397545.4	coiled-coil domain containing 40
PPM1K	ENST00000360469.5	cadherin 4, type 1, R-cadherin (retinal)
ARHGEF37	ENST00000608933.1	protein phosphatase, Mg2+/Mn2+ dependent, 1K
ZBTB7C	ENST00000333677.6	Rho guanine nucleotide exchange factor (GEF) 37
DR1	ENST00000535628.2	zinc finger and BTB domain containing 7C
DMTF1	ENST00000370272.4	down-regulator of transcription 1, TBP-binding (negative cofactor 2)
SLC25A24	ENST00000413276.2	cyclin D binding myb-like transcription factor 1
STX12	ENST00000565488.1	solute carrier family 25 (mitochondrial carrier; phosphate carrier), member 24
JARID2	ENST00000373943.4	syntaxin 12
SETD8	ENST00000341776.2	jumonji, AT rich interactive domain 2
WDR82	ENST00000402868.3	SET domain containing (lysine methyltransferase) 8
CLIP4	ENST00000296490.3	WD repeat domain 82
NUAK1	ENST00000320081.5	CAP-GLY domain containing linker protein family, member 4
KIAA2022	ENST00000261402.2	NUAK family, SNF1-like kinase, 1
ARHGAP29	ENST00000055682.6	KIAA2022
ESRRG	ENST00000260526.6	Rho GTPase activating protein 29
DEDD2	ENST00000361525.3	estrogen-related receptor gamma
SLC44A5	ENST00000336034.4	death effector domain containing 2
MAFG	ENST00000370859.3	solute carrier family 44, member 5

LAMP1	ENST00000357736.4	v-maf avian musculoaponeurotic fibrosarcoma oncogene homolog G
PRRG1	ENST00000332556.4	lysosomal-associated membrane protein 1
NR3C2	ENST00000378628.4	proline rich Gla (G-carboxyglutamic acid) 1
IPO8	ENST00000344721.4	nuclear receptor subfamily 3, group C, member 2
DPYSL3	ENST00000256079.4	importin 8
MYO10	ENST00000398514.3	dihydropyrimidinase-like 3
MGAT3	ENST00000513610.1	myosin X
FAM46A	ENST00000341184.6	mannosyl (beta-1,4-)-glycoprotein beta-1,4-N-acetylglucosaminyltransferase
FNIP1	ENST00000369754.3	family with sequence similarity 46, member A
MEIS1	ENST00000307968.7	folliculin interacting protein 1
NBEA	ENST00000444274.2	Meis homeobox 1
TFAM	ENST00000379939.2	neurobeachin
TRIM41	ENST00000487519.1	transcription factor A, mitochondrial
AKAP1	ENST00000315073.5	tripartite motif containing 41
ARL4C	ENST00000337714.3	A kinase (PRKA) anchor protein 1
CBFB	ENST00000390645.2	ADP-ribosylation factor-like 4C
ZDHHC14	ENST00000290858.6	core-binding factor, beta subunit
SPRED1	ENST00000359775.5	zinc finger, DHHC-type containing 14
BMPR1A	ENST00000299084.4	sprouty-related, EVH1 domain containing 1
ARL8B	ENST00000372037.3	bone morphogenetic protein receptor, type IA
CCNT2	ENST00000419534.2	ADP-ribosylation factor-like 8B
SPRY3	ENST00000295238.6	cyclin T2
C8orf86	ENST00000302805.2	sprouty homolog 3 (Drosophila)
KCNA3	ENST00000358138.1	chromosome 8 open reading frame 86
RBBP5	ENST00000369769.2	potassium voltage-gated channel, shaker-related subfamily, member 3
YARS	ENST00000264515.6	retinoblastoma binding protein 5

MALL	ENST00000373477.4	tyrosyl-tRNA synthetase
HMGB1	ENST00000272462.2	mal, T-cell differentiation protein-like
SCRT2	ENST00000399489.1	high mobility group box 1
MAP3K3	ENST00000246104.6	scratch homolog 2, zinc finger protein (Drosophila)
GAN	ENST00000361357.3	mitogen-activated protein kinase kinase kinase 3
WWC3	ENST00000568107.2	gigaxonin
PCYT1B	ENST00000380861.4	WWC family member 3
LIN28A	ENST00000379145.1	phosphate cytidylyltransferase 1, choline, beta
ANGPTL2	ENST00000326279.6	lin-28 homolog A (C. elegans)
EFNA5	ENST00000373425.3	angiopoietin-like 2
NEK6	ENST00000333274.6	ephrin-A5
TMED7-TICAM2	ENST00000373603.1	NIMA-related kinase 6
MRPL35	ENST00000333314.3	TMED7-TICAM2 readthrough
BCAP29	ENST00000337109.4	mitochondrial ribosomal protein L35
METAP1	ENST00000005259.4	B-cell receptor-associated protein 29
CCDC120	ENST00000296411.6	methionyl aminopeptidase 1
DGKG	ENST00000376396.3	coiled-coil domain containing 120
YIPF6	ENST00000265022.3	diacylglycerol kinase, gamma 90kDa
NR3C1	ENST00000462683.1	Yip1 domain family, member 6
DCAF16	ENST00000394464.2	nuclear receptor subfamily 3, group C, member 1 (glucocorticoid receptor)
BTBD7	ENST00000382247.1	DDB1 and CUL4 associated factor 16
SPOP	ENST00000334746.5	BTB (POZ) domain containing 7
RSRC2	ENST00000393328.2	speckle-type POZ protein
BNC2	ENST00000331738.7	arginine/serine-rich coiled-coil 2
PHF13	ENST00000380672.4	basonuclin 2
TICAM2	ENST00000377648.4	PHD finger protein 13

SH3BP5	ENST00000408996.4	toll-like receptor adaptor molecule 2
ARHGAP26	ENST00000383791.3	SH3-domain binding protein 5 (BTK-associated)
IGF2R	ENST00000378004.3	Rho GTPase activating protein 26
ELF2	ENST00000356956.1	insulin-like growth factor 2 receptor
PTPRD	ENST00000394235.2	E74-like factor 2 (ets domain transcription factor)
PPM1E	ENST00000381196.4	protein tyrosine phosphatase, receptor type, D
ELL2	ENST00000308249.2	protein phosphatase, Mg2+/Mn2+ dependent, 1E
GLIS3	ENST00000237853.4	elongation factor, RNA polymerase II, 2
TRIP12	ENST00000324333.10	GLIS family zinc finger 3
MEIS2	ENST00000283943.5	thyroid hormone receptor interactor 12
RELT	ENST00000397624.3	Meis homeobox 2
TLK1	ENST00000064780.2	RELT tumor necrosis factor receptor
MBNL1	ENST00000431350.2	tousled-like kinase 1
FBXO45	ENST00000357472.3	muscleblind-like splicing regulator 1
PDPR	ENST00000311630.6	F-box protein 45
ZNF660	ENST00000568530.1	pyruvate dehydrogenase phosphatase regulatory subunit
DUSP6	ENST00000322734.2	zinc finger protein 660
PRKCI	ENST00000279488.7	dual specificity phosphatase 6
ITPR1	ENST00000295797.4	protein kinase C, iota
NCEH1	ENST00000302640.8	inositol 1,4,5-trisphosphate receptor, type 1
ABCG8	ENST00000475381.1	neutral cholesterol ester hydrolase 1
BCL9	ENST00000272286.2	ATP-binding cassette, sub-family G (WHITE), member 8
FBXO9	ENST00000234739.3	B-cell CLL/lymphoma 9
METTL7A	ENST00000244426.6	F-box protein 9
AGPAT4	ENST00000332160.4	methyltransferase like 7A
RALGPS2	ENST00000366911.5	1-acylglycerol-3-phosphate O-acyltransferase 4

PPTC7	ENST00000367635.3	Ral GEF with PH domain and SH3 binding motif 2
DCAF8	ENST00000354300.3	PTC7 protein phosphatase homolog (S. cerevisiae)
FRAS1	ENST00000368073.3	DDB1 and CUL4 associated factor 8
TPPP	ENST00000264895.6	Fraser syndrome 1
MXI1	ENST00000360578.5	tubulin polymerization promoting protein
PPP1CC	ENST00000332674.5	MAX interactor 1, dimerization protein
DLG5	ENST00000335007.5	protein phosphatase 1, catalytic subunit, gamma isozyme
GRIN2B	ENST00000372391.2	discs, large homolog 5 (Drosophila)
TMEM86A	ENST00000609686.1	glutamate receptor, ionotropic, N-methyl D-aspartate 2B
CPOX	ENST00000556710.1	DDB1- and CUL4-associated factor 8
RIN3	ENST00000280734.2	transmembrane protein 86A
CDH2	ENST00000264193.2	coproporphyrinogen oxidase
BDNF	ENST00000216487.7	Ras and Rab interactor 3
PCGF3	ENST00000269141.3	cadherin 2, type 1, N-cadherin (neuronal)
KHDRBS1	ENST00000439476.2	brain-derived neurotrophic factor
C20orf194	ENST00000362003.5	polycomb group ring finger 3
RRM2	ENST00000327300.7	KH domain containing, RNA binding, signal transduction associated 1
FAM117B	ENST00000453730.2	chromosome 20 open reading frame 194
ADAMTS9	ENST00000360566.2	ribonucleotide reductase M2
P2RX7	ENST00000392238.2	family with sequence similarity 117, member B
ERGIC1	ENST00000295903.4	ADAM metallopeptidase with thrombospondin type 1 motif, 9
POLR2F	ENST00000328963.5	purinergic receptor P2X, ligand-gated ion channel, 7
GABBR2	ENST00000393784.3	endoplasmic reticulum-golgi intermediate compartment (ERGIC) 1
ZDHHC17	ENST00000442738.2	polymerase (RNA) II (DNA directed) polypeptide F
PPARGC1A	ENST00000259455.2	gamma-aminobutyric acid (GABA) B receptor, 2
INSM1	ENST00000426126.2	zinc finger, DHHC-type containing 17

STOX2	ENST00000264867.2	peroxisome proliferator-activated receptor gamma, coactivator 1 alpha
ADCY6	ENST00000310227.1	insulinoma-associated 1
ZFHX3	ENST00000308497.4	storkhead box 2
MPZ	ENST00000357869.3	adenylate cyclase 6
PLXNA2	ENST00000268489.5	zinc finger homeobox 3
FAM126A	ENST00000533357.1	myelin protein zero
NRG3	ENST00000367033.3	plexin A2
BIRC6	ENST00000409923.1	family with sequence similarity 126, member A
BHLHE22	ENST00000372142.2	neuregulin 3
RICTOR	ENST00000421745.2	baculoviral IAP repeat containing 6
FLT3	ENST00000321870.1	basic helix-loop-helix family, member e22
ZNF335	ENST00000357387.3	RPTOR independent companion of MTOR, complex 2
SEC31B	ENST00000241453.7	fms-related tyrosine kinase 3
ZNRF3	ENST00000322927.2	zinc finger protein 335
GABRB3	ENST00000370345.3	SEC31 homolog B (S. cerevisiae)
BCL11B	ENST00000544604.2	zinc and ring finger 3
SGCZ	ENST00000311550.5	gamma-aminobutyric acid (GABA) A receptor, beta 3
MEX3A	ENST00000357195.3	B-cell CLL/lymphoma 11B (zinc finger protein)
RASSF5	ENST00000382080.1	sarcoglycan, zeta
DBH	ENST00000532414.2	mex-3 RNA binding family member A
SATB2	ENST00000367117.3	Ras association (RalGDS/AF-6) domain family member 5
ZBTB7A	ENST00000393056.2	dopamine beta-hydroxylase (dopamine beta-monooxygenase)
KIAA1324L	ENST00000417098.1	SATB homeobox 2
PEG10	ENST00000322357.4	zinc finger and BTB domain containing 7A
ANKFY1	ENST00000450689.2	KIAA1324-like
FAM83F	ENST00000482108.1	paternally expressed 10

GXYLT2	ENST00000341657.4	ankyrin repeat and FYVE domain containing 1
IRF2BP2	ENST00000333407.6	family with sequence similarity 83, member F
ELAVL4	ENST00000389617.4	glucoside xylosyltransferase 2
RAB10	ENST00000366610.3	interferon regulatory factor 2 binding protein 2
ZBTB8B	ENST00000371824.1	ELAV like neuron-specific RNA binding protein 4
B3GNT1	ENST00000264710.4	RAB10, member RAS oncogene family
ZNF282	ENST00000609129.1	zinc finger and BTB domain containing 8B
THBS2	ENST00000311181.4	UDP-GlcNAc:betaGal beta-1,3-N-acetylglucosaminyltransferase 1
FRMD5	ENST00000479907.1	zinc finger protein 282
GCC1	ENST00000366787.3	thrombospondin 2
BRD4	ENST00000484674.1	FERM domain containing 5
DNAJB1	ENST00000321407.2	GRIP and coiled-coil domain containing 1
CAPN14	ENST00000263377.2	bromodomain containing 4
RAB14	ENST00000254322.2	DnaJ (Hsp40) homolog, subfamily B, member 1
INPP4A	ENST00000444918.2	calpain 14
TNFRSF8	ENST00000373840.4	RAB14, member RAS oncogene family
EPC2	ENST00000409016.4	inositol polyphosphate-4-phosphatase, type I, 107kDa
BRPF3	ENST00000263932.2	tumor necrosis factor receptor superfamily, member 8
MBTPS2	ENST00000258484.6	enhancer of polycomb homolog 2 (Drosophila)
GPM6A	ENST00000357641.6	bromodomain and PHD finger containing, 3
TRPM3	ENST00000379484.5	membrane-bound transcription factor peptidase, site 2
TCF7L1	ENST00000280187.7	glycoprotein M6A
GAPVD1	ENST00000377111.2	transient receptor potential cation channel, subfamily M, member 3
GPT2	ENST00000282111.3	transcription factor 7-like 1 (T-cell specific, HMG-box)
BTAF1	ENST00000470056.1	GTPase activating protein and VPS9 domains 1
CAPRIN1	ENST00000340124.4	glutamic pyruvate transaminase (alanine aminotransferase) 2

DIS3	ENST00000265990.6	BTAF1 RNA polymerase II, B-TFIID transcription factor-associated, 170kDa
ZNF423	ENST00000341394.4	cell cycle associated protein 1
FUBP3	ENST00000377767.4	DIS3 mitotic control homolog (S. cerevisiae)
IPO5	ENST00000561648.1	zinc finger protein 423
SLC38A9	ENST00000319725.9	far upstream element (FUSE) binding protein 3
MYT1L	ENST00000261574.5	importin 5
SLC17A7	ENST00000396865.2	solute carrier family 38, member 9
CNTNAP2	ENST00000399161.2	myelin transcription factor 1-like
NRP1	ENST00000221485.3	solute carrier family 17 (vesicular glutamate transporter), member 7
SLITRK4	ENST00000361727.3	contactin associated protein-like 2
PPP6C	ENST00000374875.1	neuropilin 1
SMC2	ENST00000381779.4	SLIT and NTRK-like family, member 4
ADAMTS5	ENST00000373547.4	protein phosphatase 6, catalytic subunit
DLG1	ENST00000374793.3	structural maintenance of chromosomes 2
CDK13	ENST00000284987.5	ADAM metallopeptidase with thrombospondin type 1 motif, 5
DENR	ENST00000346964.2	discs, large homolog 1 (Drosophila)
CTDSPL2	ENST00000181839.4	cyclin-dependent kinase 13
GPBP1L1	ENST00000280557.6	density-regulated protein
HDAC9	ENST00000260327.4	CTD (carboxy-terminal domain, RNA polymerase II, polypeptide A) small phosphatase like 2
PIP4K2B	ENST00000290795.3	GC-rich promoter binding protein 1-like 1
CELSR3	ENST00000405010.3	histone deacetylase 9
NOS1AP	ENST00000269554.3	phosphatidylinositol-5-phosphate 4-kinase, type II, beta
SIM1	ENST00000164024.4	cadherin, EGF LAG seven-pass G-type receptor 3
ZCCHC14	ENST00000361897.5	nitric oxide synthase 1 (neuronal) adaptor protein
PPP1R9A	ENST00000369208.3	single-minded homolog 1 (Drosophila)
THSD4	ENST00000268616.4	zinc finger, CCHC domain containing 14

TIAL1	ENST00000289495.5	protein phosphatase 1, regulatory subunit 9A
LGR4	ENST00000355327.3	thrombospondin, type I, domain containing 4
CDH11	ENST00000369093.2	TIA1 cytotoxic granule-associated RNA binding protein-like 1
KIAA0226	ENST00000379214.4	leucine-rich repeat containing G protein-coupled receptor 4
KCNC2	ENST00000394156.3	cadherin 11, type 2, OB-cadherin (osteoblast)
WDFY2	ENST00000273582.5	KIAA0226
ZFHX4	ENST00000548513.1	potassium voltage-gated channel, Shaw-related subfamily, member 2
PRPF38B	ENST00000298125.5	WD repeat and FYVE domain containing 2
PLAA	ENST00000521891.2	zinc finger homeobox 4
WNT4	ENST00000370025.4	pre-mRNA processing factor 38B
SRPK2	ENST00000397292.3	phospholipase A2-activating protein
CIAO1	ENST00000290167.6	wingless-type MMTV integration site family, member 4
NPAS3	ENST00000393651.3	SRSF protein kinase 2
TAOK1	ENST00000488633.1	cytosolic iron-sulfur protein assembly 1
MMAA	ENST00000346562.2	neuronal PAS domain protein 3
TP53INP1	ENST00000261716.3	TAO kinase 1
SHC1	ENST00000281317.5	methylmalonic aciduria (cobalamin deficiency) cblA type
TFAP2B	ENST00000448464.2	tumor protein p53 inducible nuclear protein 1
ATP2B1	ENST00000368445.5	SHC (Src homology 2 domain containing) transforming protein 1
SP6	ENST00000393655.3	transcription factor AP-2 beta (activating enhancer binding protein 2 beta)
TFAP2A	ENST00000261173.2	ATPase, Ca++ transporting, plasma membrane 1
TMEM194A	ENST00000342234.2	Sp6 transcription factor
CDC73	ENST00000379613.3	transcription factor AP-2 alpha (activating enhancer binding protein 2 alpha)
HSPA13	ENST00000379391.3	transmembrane protein 194A
XPO5	ENST00000367435.3	cell division cycle 73
NIPBL	ENST00000285667.3	heat shock protein 70kDa family, member 13

HMGCR	ENST00000265351.7	exportin 5
ATXN7L1	ENST00000448238.2	Nipped-B homolog (Drosophila)
CASP9	ENST00000287936.4	3-hydroxy-3-methylglutaryl-CoA reductase
ZNF462	ENST00000419735.3	ataxin 7-like 1
GSTM3	ENST00000546424.1	caspase 9, apoptosis-related cysteine peptidase
KCNA1	ENST00000277225.5	zinc finger protein 462
DNMBP	ENST00000540225.1	glutathione S-transferase mu 3 (brain)
SCN4A	ENST00000382545.3	potassium voltage-gated channel, shaker-related subfamily, member 1 (episodic ataxia with myokymia)
UNC5B	ENST00000324109.4	dynamin binding protein
NRARP	ENST00000578147.1	sodium channel, voltage-gated, type IV, alpha subunit
KLHL13	ENST00000335350.6	unc-5 homolog B (C. elegans)
RANBP2	ENST00000356628.2	NOTCH-regulated ankyrin repeat protein
M6PR	ENST00000371882.1	kelch-like family member 13
DDX18	ENST00000283195.6	RAN binding protein 2
FARP1	ENST00000000412.3	mannose-6-phosphate receptor (cation dependent)
TANC2	ENST00000263239.2	DEAD (Asp-Glu-Ala-Asp) box polypeptide 18
ATP8B1	ENST00000595437.1	FERM, RhoGEF (ARHGEF) and pleckstrin domain protein 1 (chondrocyte-derived)
BAZ2A	ENST00000424789.2	tetratricopeptide repeat, ankyrin repeat and coiled-coil containing 2
MRPL17	ENST00000283684.4	ATPase, aminophospholipid transporter, class I, type 8B, member 1
PRDM6	ENST00000379441.3	bromodomain adjacent to zinc finger domain, 2A
ITGA11	ENST00000288937.6	mitochondrial ribosomal protein L17
ELMOD3	ENST00000407847.4	PR domain containing 6
SIN3A	ENST00000423218.2	integrin, alpha 11
MSL2	ENST00000315658.7	ELMO/CED-12 domain containing 3
ARHGAP5	ENST00000394947.3	SIN3 transcription regulator family member A
CPD	ENST00000309993.2	male-specific lethal 2 homolog (Drosophila)

RERE	ENST00000345122.3	Rho GTPase activating protein 5
SOCS7	ENST00000225719.4	carboxypeptidase D
GSPT1	ENST00000337907.3	arginine-glutamic acid dipeptide (RE) repeats
ANXA11	ENST00000577233.1	suppressor of cytokine signaling 7
PERP	ENST00000434724.2	G1 to S phase transition 1
RBM24	ENST00000372231.3	annexin A11
CUL4B	ENST00000421351.3	PERP, TP53 apoptosis effector
EEF1E1	ENST00000379052.5	RNA binding motif protein 24
TRIM13	ENST00000371322.5	cullin 4B
C6orf57	ENST00000379715.5	eukaryotic translation elongation factor 1 epsilon 1
POLA1	ENST00000378182.3	tripartite motif containing 13
CCPG1	ENST00000370474.3	chromosome 6 open reading frame 57
ATG7	ENST00000379068.3	polymerase (DNA directed), alpha 1, catalytic subunit
ANKH	ENST00000310958.6	cell cycle progression 1

Supplementary Table 4b. 78 common target genes in "Targetscan", "miRanda", "miRWalk" and "PBMCs Up"

name	p (Corr)	FC (abs)	Representative transcript	Gene name
ADD3	0.000101607	2.118956636	ENST00000277900.8	adducin 3 (gamma)
AKAP2	4.01408E-05	3.660171262	ENST00000374525.1	A kinase (PRKA) anchor protein 2
ANKRD12	0.010871936	1.746766905	ENST00000262126.4	ankyrin repeat domain 12
ANKRD13A	0.001786033	1.752628754	ENST00000261739.4	ankyrin repeat domain 13A
AP1S2	3.4323E-05	2.526598093	ENST00000329235.2	adaptor-related protein complex 1, sigma 2 subunit
ARAP2	0.000461136	1.550532545	ENST00000303965.4	ArfGAP with RhoGAP domain, ankyrin repeat and PH domain 2
ARHGEF33	0.00331942	3.617750948	ENST00000409978.1	Rho guanine nucleotide exchange factor (GEF) 33
ARL5A	0.000341597	2.175050123	ENST00000295087.8	ADP-ribosylation factor-like 5A
ARL5B	3.13704E-05	1.669671847	ENST00000377275.3	ADP-ribosylation factor-like 5B

ATXN1	0.025681898	1.819142251	ENST00000244769.4	ataxin 1
BCL2	3.628E-05	2.443430334	ENST00000398117.1	B-cell CLL/lymphoma 2
BNIP2	2.77739E-05	2.535041633	ENST00000267859.3	BCL2/adenovirus E1B 19kDa interacting protein 2
CCDC6	0.000131838	2.133943751	ENST00000263102.6	coiled-coil domain containing 6
CCNDBP1	6.85374E-05	1.724815568	ENST00000300213.4	cyclin D-type binding-protein 1
CHN2	0.000292284	2.102503525	ENST00000222792.6	chimerin 2
CORO1C	6.89884E-05	2.212787287	ENST00000261401.3	coronin, actin binding protein, 1C
COX5A	0.000207274	2.471821946	ENST00000322347.6	cytochrome c oxidase subunit Va
CREB1	7.13985E-05	2.141457017	ENST00000432329.2	cAMP responsive element binding protein 1
CREB5	0.000144076	2.453704476	ENST00000357727.2	cAMP responsive element binding protein 5
CRKL	0.00024704	2.103208476	ENST00000354336.3	v-crk avian sarcoma virus CT10 oncogene homolog-like
DCUN1D3	0.016404562	1.659690549	ENST00000324344.4	DCN1, defective in cullin neddylation 1, domain containing 3
DYRK1A	0.000134938	2.225386189	ENST00000339659.4	dual-specificity tyrosine-(Y)-phosphorylation regulated kinase 1A
EAF1	0.000670659	2.192433004	ENST00000396842.2	ELL associated factor 1
ERBB2IP	0.000120039	1.93163232	ENST00000284037.5	erbb2 interacting protein
FAM107B	1.10614E-05	2.018470168	ENST00000378470.1	family with sequence similarity 107, member B
FAM168B	0.000217065	2.043272025	ENST00000409185.1	family with sequence similarity 168, member B
FAM175B	0.001263133	1.992051922	ENST00000298492.5	family with sequence similarity 175, member B
FBXW7	0.000333319	1.797062159	ENST00000281708.4	F-box and WD repeat domain containing 7, E3 ubiquitin protein ligase
FOXK2	0.003763641	1.668985977	ENST00000335255.5	forkhead box K2
HELZ	6.04888E-05	1.876082237	ENST00000358691.5	helicase with zinc finger
LEMD3	0.000765056	1.563030072	ENST00000308330.2	LEM domain containing 3
LMOD3	0.003459176	1.597812874	ENST00000420581.2	leiomodin 3 (fetal)
LRRC55	0.000122135	1.961164509	ENST00000497933.1	leucine rich repeat containing 55
MAN1A2	0.005345608	1.613402178	ENST00000356554.3	mannosidase, alpha, class 1A, member 2
MAP1LC3B	0.005120067	1.64351389	ENST00000268607.5	microtubule-associated protein 1 light chain 3 beta

MARCKS	0.001388651	1.808312208	ENST00000368635.4	myristoylated alanine-rich protein kinase C substrate
MLLT3	0.004894843	1.649597889	ENST00000380338.4	myeloid/lymphoid or mixed-lineage leukemia (trithorax homolog, Drosophila); translocated to, 3
MON2	1.99772E-05	1.802661836	ENST00000546600.1	MON2 homolog (S. cerevisiae)
NOTCH2	0.000135872	2.593497844	ENST00000256646.2	notch 2
OGT	0.000478561	1.573514099	ENST00000373719.3	O-linked N-acetylglucosamine (GlcNAc) transferase
PLXDC2	0.000225904	2.03640044	ENST00000377252.4	plexin domain containing 2
PRR11	0.003914933	1.614611607	ENST00000262293.4	proline rich 11
PSME1	0.000439239	1.708774745	ENST00000382708.3	proteasome (prosome, macropain) activator subunit 1 (PA28 alpha)
PTPRJ	0.000343228	2.343191954	ENST00000418331.2	protein tyrosine phosphatase, receptor type, J
RAB1A	0.001395771	2.08416751	ENST00000409892.1	RAB1A, member RAS oncogene family
RABGAP1L	0.000184767	2.060806026	ENST00000489615.1	RAB GTPase activating protein 1-like
RCOR1	0.000106394	1.671716927	ENST00000262241.6	REST corepressor 1
RHOT1	0.001646125	1.642465336	ENST00000333942.6	ras homolog family member T1
SEC24D	0.000949566	1.57932802	ENST00000429811.2	SEC24 family, member D (S. cerevisiae)
SLC16A6	1.77867E-05	1.753766113	ENST00000327268.4	solute carrier family 16, member 6
SLC37A3	0.00017147	1.982247081	ENST00000447932.2	solute carrier family 37, member 3
SLC39A11	6.24855E-06	2.098699859	ENST00000255559.3	solute carrier family 39, member 11
SORT1	0.003458671	1.721344321	ENST00000256637.6	sortilin 1
TCF12	0.000807178	1.695698172	ENST00000267811.5	transcription factor 12
TET2	0.000662609	1.89988674	ENST00000545826.1	tet methylcytosine dioxygenase 2
TFEC	0.021407971	1.913632445	ENST00000265440.7	transcription factor EC
TGFBR1	0.001039909	2.109706618	ENST00000374994.4	transforming growth factor, beta receptor 1
TMEM181	0.000671323	1.662792711	ENST00000367090.3	transmembrane protein 181
TMEM218	8.5339E-05	1.991904215	ENST00000532156.1	transmembrane protein 218
TMEM64	0.000403021	1.650260063	ENST00000458549.2	transmembrane protein 64
TOMM70A	0.000671886	1.947860837	ENST00000284320.5	translocase of outer mitochondrial membrane 70 homolog A (S. cerevisiae)

TPM3	0.041852441	1.550165352	ENST00000368531.2	tropomyosin 3
UBA2	0.001691854	1.630646397	ENST00000246548.4	ubiquitin-like modifier activating enzyme 2
UBA6	0.000195712	1.672938819	ENST00000322244.5	ubiquitin-like modifier activating enzyme 6
UBP1	0.000100507	1.843642401	ENST00000283629.3	upstream binding protein 1 (LBP-1a)
USP47	0.000140434	1.731478309	ENST00000339865.5	ubiquitin specific peptidase 47
VAPB	0.001333989	2.099252037	ENST00000475243.1	VAMP (vesicle-associated membrane protein)-associated protein B and C
VEZT	0.00010095	2.409261345	ENST00000436874.1	vezatin, adherens junctions transmembrane protein
WDFY3	0.000122223	2.137728303	ENST00000322366.6	WD repeat and FYVE domain containing 3
WEE1	2.50671E-05	2.400540012	ENST00000299613.6	WEE1 homolog (S. pombe)
WNK1	0.000118926	1.710329219	ENST00000315939.6	WNK lysine deficient protein kinase 1
XRN1	0.000205849	1.596227694	ENST00000264951.4	5'-3' exoribonuclease 1
YWHAZ	0.000222343	1.594285273	ENST00000395957.2	tyrosine 3-monooxygenase/tryptophan 5-monooxygenase activation protein, zeta polypeptide
ZCCHC11	4.8572E-05	2.793995981	ENST00000257177.4	zinc finger, CCHC domain containing 11
ZFP91	7.38433E-05	2.063744866	ENST00000316059.6	ZFP91 zinc finger protein
ZNF146	0.000874062	2.217490363	ENST00000443387.2	zinc finger protein 146
ZNF638	0.001720886	1.664544601	ENST00000355812.3	zinc finger protein 638
ZNF654	0.000267207	2.785386266	ENST00000309495.5	zinc finger protein 654

Reference

1 Zhu, H. *et al.* Rheumatoid arthritis-associated DNA methylation sites in peripheral blood mononuclear cells. *Ann Rheum Dis* **78**, 36-42 (2019).
